# Beyond air temperature: An examination of leveraging operative temperature space for the discovery of bioinspired thermal design features

**DOI:** 10.1371/journal.pone.0358463

**Published:** 2026-09-16

**Authors:** Thomas N. Slavens, Hamid Shabgard

**Affiliations:** College of Aerospace and Mechanical Engineering, University of Oklahoma, Norman, Oklahoma, United States of America; Manipal Academy of Higher Education, INDIA

## Abstract

Advancements in thermal management systems often rely on incorporating novel design features which can be effectively influenced through bioinspiration. A major gap in applying bioinspired concepts to thermal design is the identification of morphological structures of interest due to the multimodal nature of most energy transfer systems. Investigating species along an operative temperature gradient—which incorporates convective and radiative heat transfer—offers a more comprehensive view of environmental heat load than traditional ambient air temperature or latitude, providing better granularity to identify phenotypic plasticity driven by heat load. This study refined a thermally oriented bioinspiration search method by clustering species spatial group locations based on growing season operative temperature distributions. Focusing on the North American tree species *Populus heterophylla*, thermal clines were developed using a density-based cluster analysis utilizing ERA5-Land climate data to evaluate both 2-meter air temperatures and calculated operative temperatures. To evaluate phenotypic trends across these thermal regimes, 2D leaf margins from 103 herbarium specimens were analyzed for aspect ratio, leaf width, and non-dimensionalized characteristic edge radius. While ambient air temperature clustering yielded only two broad regional groups, operative temperature mapping provided greater environmental granularity, resolving four distinct spatial groups, including unique mid-latitude and coastal sub-populations. Specimen analysis revealed that cooler-climate groups exhibited smaller edge radii (higher margin dissection) than warmer-climate groups, with intermediate clusters displaying transitional values between the extreme ranges. These results demonstrate that operative temperature space offers an improved framework for identifying candidate morphological features associated with regional thermal environments, laying a foundation to test potential heat-transfer adaptations for engineering design.

## Introduction

Advancements in several fields rely heavily on the ability to develop highly efficient means to thermally manage components. Gas turbines depend greatly on convective cooling techniques to keep hot section components from rapidly deteriorating typically leveraging surface film-cooling holes [1] or internal channels utilizing turbulence promoters [2,3]. The design of thermal systems at scale typically leverages corporate engineering knowledge systems which heavily rely on standardized processes that allow for enterprise execution of engineering work. These systems are built on the state-of-art processes and features of their inception, but typically lack agility to pivot with new technologies that can increase thermal design space. Disruptive technologies such as additive manufacturing or metal injection molding can go under-utilized during the concept phase of product development due to lack of feature sets readily available at designers hands. It is then advantageous to have a means to quickly search for inspirations for new features that support the design from first principles [4].

As described in previous work [5], *bioinspiration* can be leveraged to inject novel concepts into design work. *Bioinspiration* utilizes biologically-inspired mechanisms for use in solutions adjacent to the intent of the adaptation [6]. It has been found to be an effective means to generate unique design intuitions based on correlation of ideas between the problem being addressed and observed solutions in nature [7]. A major hurdle to readily leveraging bioinspiration is the need to draw information across disparate disciplines at the point of concept initiation where it is most impactful [8,9].

To close this gap with respect to thermal design, previous work has sought to develop a search strategy to highlight species of interest for study based on heat load exposure along a thermal cline [5]. This work derived an operative temperature parameter based on a massless leaf in heat balance with the environment providing a spatially relevant metric that incorporates convective and radiative modes of heat transfer. This *operative temperature* parameter was calculated across the Continental United States (CONUS) utilizing ERA5-Land climate data averaged from January 1970 to December 2000 and applied to the basal ranges of tree species of the region. The species were then ranked based on the range of maximum operative temperature the species encounters across their largest contiguous spatial distribution. Correlation assessments comparing average leaf size trends to operative temperature was conducted to identify if this parameter offered a stronger signal to morphological variation than typically used air temperature or latitude factors. The assessment showed an extension to the parameter range that the species saw, but did not provide a meaningfully stronger signal in highlighting leaf characteristic differences than ambient temperature or latitude [5]. This result pointed out that deeper functional traits and features, such as leaf margin variation, may be factors more aligned with temperature driven species adaption.

Studies have observed positive increase to the number of species that exhibit roughening at the margin of their leaves, vascularized projections often observed as serration, with decrease in mean annual temperature [10–14]. This dynamic has also been observed from within-species margin variation from taxa grown in warm climates compared to those grown in cooler climates [15,16]. Experimental testing of heat transfer variation on leaf models showed increase of convection rates with leaf tooth size [17]. Wind tunnel assessments of boundary layer turbulence have shown a reduction in critical Reynolds number with leaves having a crenate margin compared to a flat plate model [18].The teething is hypothesized to benefit rates of carbon uptake and water exchange, both of which is beneficial in cooler climates boosting early-stage development but are a detriment in warmer and more arid regions [19]. While these results indicate this morphology benefits rates of convective transfer from a leaf, further study of how these features coincide with more granular environmental and geometric parameters is necessary to understand the mechanisms that frame the biological problem being addressed.

The heat-load driven operative temperature estimates a larger thermal exposure range compared to ambient temperatures and latitudes due to inclusion of solar heating during the day and radiative and convective losses at night [5]. This extension of parameter space may provide a means to find meaningful species sub-populations through differentiation of inter-species groups that reside in particular heat load spaces. This parameter space extension may also provide the necessary granularity to better contextualize adaptive features such as leaf margin serration. The current study leverages the previously derived operative temperature metric to develop a method to identify intra-species group differences based on both air and operative temperature space segregation focusing on leaf margin variation of a specific tree species within the continental United States: *Populus heterophylla*. The goal of this study is to (1) provide an updated thermally-centric bioinspiration search method utilizing operative temperature distributions to identify pertinent intra-species groups under distinctly different heat loading and (2) identify leaf margin trends under typically used ambient temperatures versus the proposed operative temperature metric.

The approach developed in this work demonstrates a framework to investigate species for thermally adaptive structures of interest based on their phenotypic response under differing environmental heat load. Leveraging this operative temperature provides a means to study species thermal plasticity based on total environmental heat load beyond typical latitude or air temperature-driven abiotic parameters without treating related thermal conditions independently. The study focuses on two results of leveraging this parameter: (1) clustering results investigating operative temperature driven clustering groups and (2) the ability to discern differences of interest in leaf margins of the study species.

## Methods

### Species subgroup development based on temperature space

**Fig 1:**
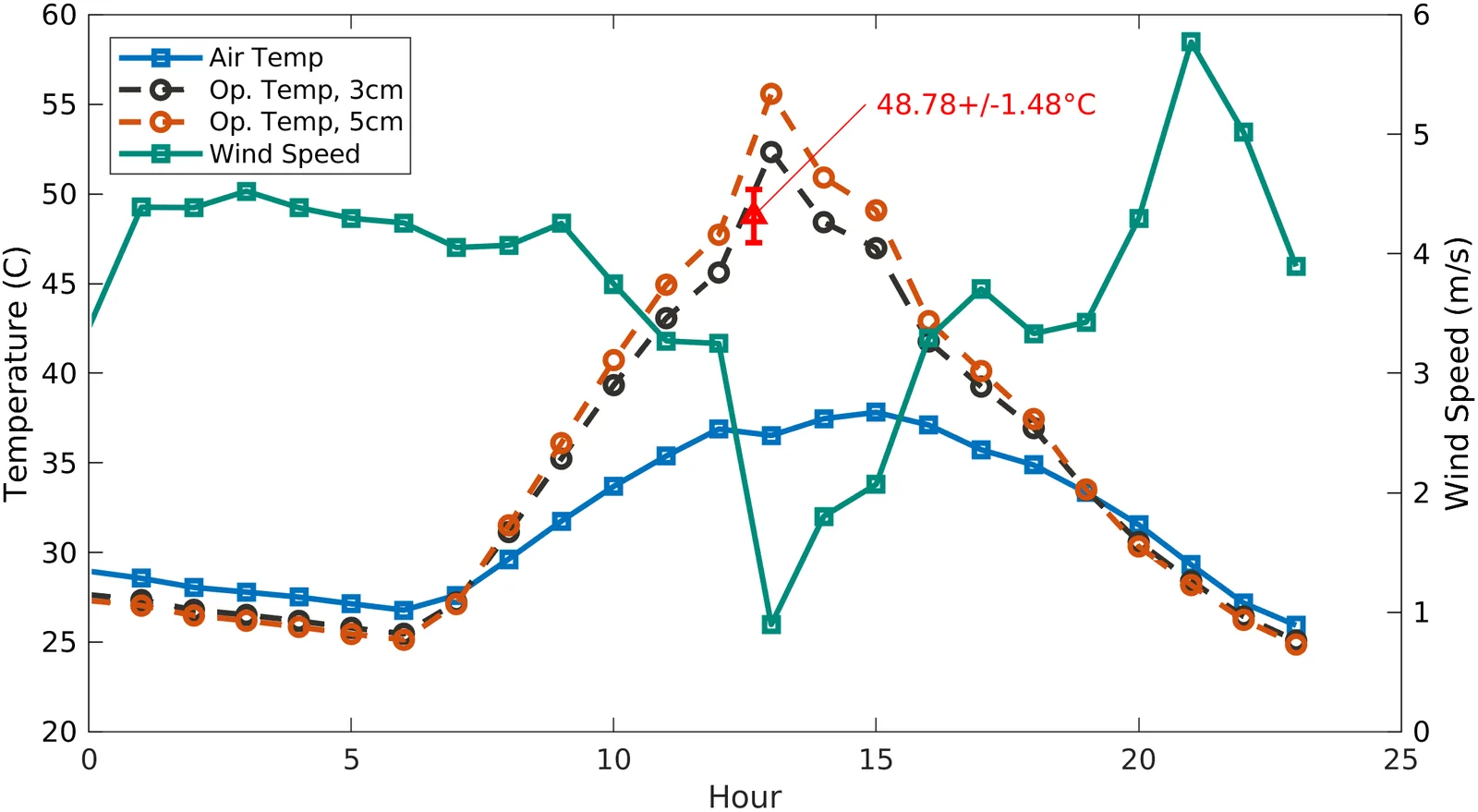
Example of ERA5 Land parameters and operative temperature calculations. 2-m air temperatures (blue), wind speed (green), and operative temperature estimates (3 cm surface in black, 5 cm surface in orange) over a diurnal time period for a test location during July 30^th^, 2025 (Location = 36.4° Latitude, −98.6° Longitude). IR thermal measurements of a black 3 cm square construction paper model were taken on this day identified by the red triangle. The paper model (emissivity assumed to be 0.94 [21], reflectivity assumed to be 0.4 [22]) was leveraged to assess in-field performance of a low-mass physical model to the operative temperature estimates. The operative temperature estimate trend was found to coincide with the physical model having the hourly trends within the 95% uncertainty interval of the measurements.

A thermal metric incorporating solar radiation, convection, and radiative emission is leveraged as the basis to calculate an *operative temperature* that represents the heat exposure of a species [5]. This calculation utilizes ERA5-Land data from January 1970 to December 2000 on a 0.08° x 0.08° degree grid (representing a grid size of roughly 9 km equatorially) on an hourly basis to calculate the operative temperature based on a 3 cm wide massless surface across the Continental United States (CONUS) region [5]. The ERA5-Land dataset leverages atmospheric reanalysis data in driving a land surface model which calculates energy fluxes at the surface [20]. Utilizing this dataset, the operative temperature is calculated in every grid cell on an hourly basis over the dataset time range stated above. An example of the calculated operative temperature over a 24 hour period is shown in Fig 1 compared to an infield observation from a low-mass physical model. The maximum, minimum, and average operative temperatures are then calculated for each month as averaged across the thirty year study period. These operative temperatures are then mapped to tree species basal areas [23] producing a spatially driven average operative temperature range.

**Fig 2:**
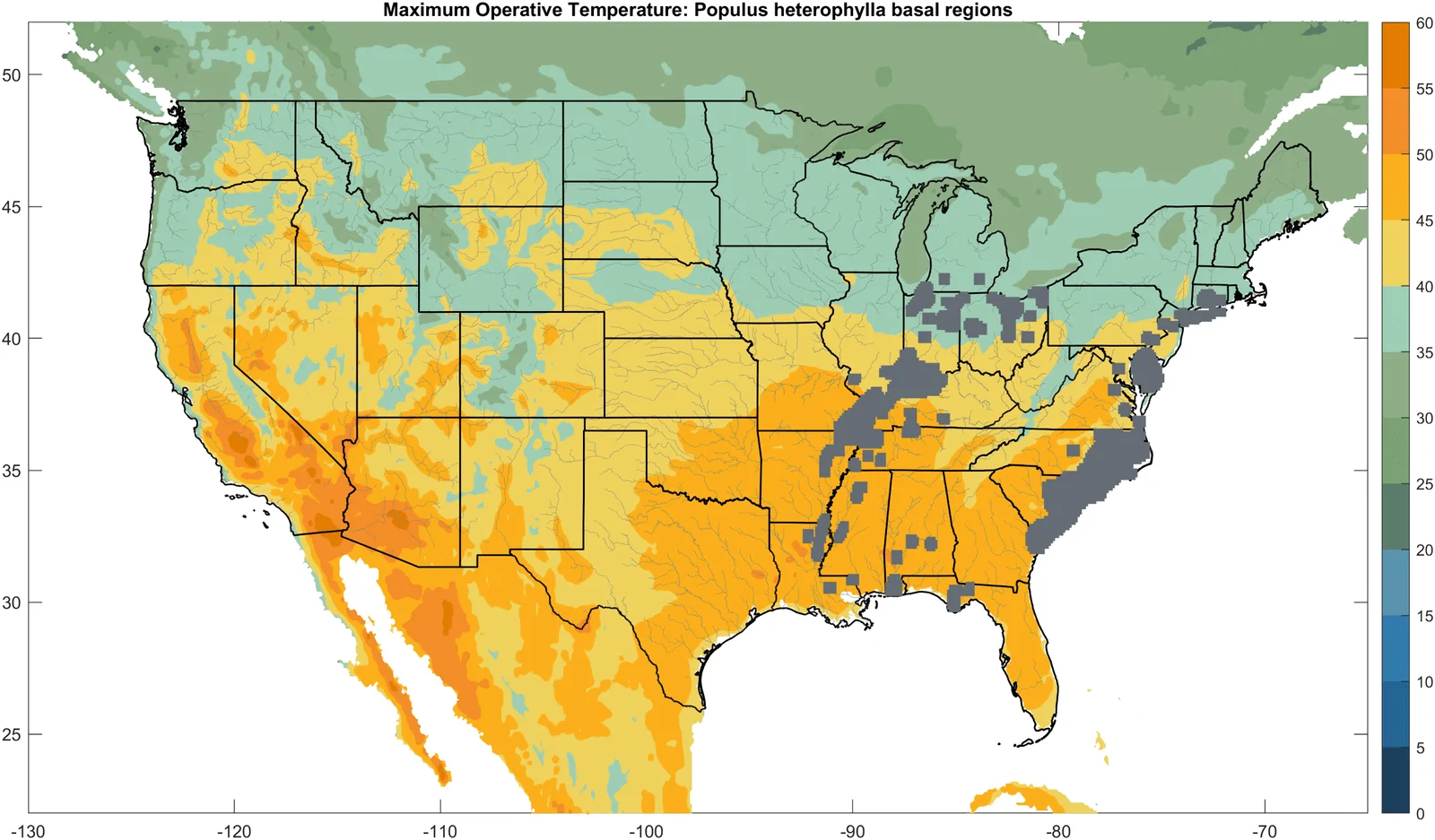
Basal range of P. heterophylla (grey markers) overlaid on the maximum operative temperature (°C) for July. Basal region occurs in pockets through the study region over varying operative temperature regimes.

This operative temperature range was then used to rank the 679 tree species in the CONUS study region ranking them from large range to those of smaller range. From this list a species of interest, P. heterophylla was identified that had spatially disbursed species pockets. Fig 2 shows the basal range of P. heterophylla plotted on the maximum operative temperature map for the month of July. The discrete pockets of species occurrence highlight possible locations where variations in operative temperature space may drive phenotypic morphological variations that may be meaningful for a thermal designer.

The basal distribution of the study species was then broken down into different spatial groupings representing different heat load environments identified using a density-based cluster analysis in temperature space. The cluster analysis leverages the maximum, minimum, and average monthly temperatures from March (month 3) to October (month 10) relating to early and late growing seasons in the CONUS region of study.

The cluster analysis utilized a density-based spatial clustering application with noise (DBSCAN) [24]. Sensitivity evaluation of the neighborhood search radius (ε) was conducted independently between both air temperature and operative temperature space. This sensitivity study was developed over a sweep of radii and minimum neighborhood populations to determine where 90% of the spatial points were captured and the average cluster sizes stabilized. This assessment was completed for both operative temperatures and for the ERA5-Land T2M parameter representing the 2 meter air temperature of the grid point.

### Study specimens from digital herbarium

Efforts to digitize and provide online herbarium specimens has been underway over the past decades [25,26]. These collections are composed of digitized type specimens that typically include taxa identification, classification, collection date, and collection location [25]. The emergence of online herbarium databases have allowed for a number of studies ranging from updated geographic species ranges [25], studies in changes to phenology of species [27,28], invasive species growth [29], and impacts of climate change to species [30]. Herbarium specimens have also been used to provide filling samples to morphology variation studies [31–33].

While the use of herbarium specimens provide a meaningful tranche of data to sample from, a number of limitations impact the data sets. Biases in herbaria data have been noted to be (1) meta-data errors of samples, (2) high variability in sampling over a long period of time, (3) timing of seasons of when the samples were taken, (4) oversampling of species at a certain locations, and (5) limitation on broad plant condition due to only a small portion of the plant collected [28,29,34]. The impacts of these biases vary upon the aims of the study being conducted, but techniques have been developed to assess and minimize downstream errors [26,32,34].

**Fig 3:**
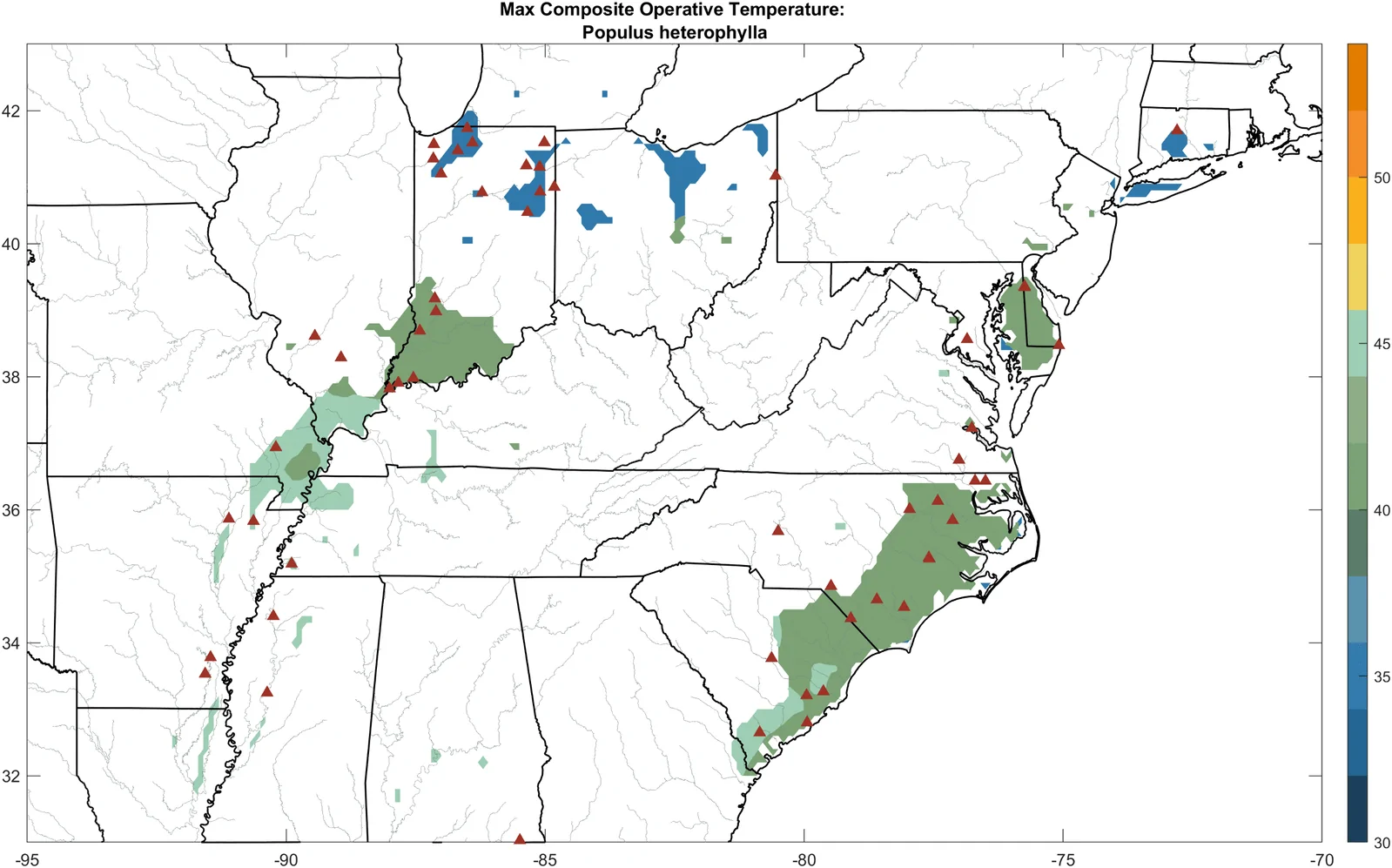
Distribution of digital specimens (denoted by red triangles) collected for P. heterophylla spanning the basal distribution of the species highlighted by location maximum operative temperature.

The current study utilizes 103 leaf specimens obtained from online herbarium databases within the study region [35–37]. These were down-selected after an initial review of samples in the study region. Specimens were selected based on visual completeness of the leaf margin (>90%). Of the 103 digitized specimens, a subset was selected with a leaf width range of greater than 5.5 representing mature leaves [38]. To minimize the impacts of location oversampling, specimens were collected broadly across the distribution range of the species as shown in Fig 3. Further, leaves were selected across the growing season to ensure that mature specimens were taken into consideration. While date criteria, range sampling, and size criteria were taken to minimize biases (3), (4) and (5) identified above, the extended climatic timeframe the temperatures were developed from seeks to couch the spatial morphological comparisons on long-term climatological basis rather than seasonal exposure outcomes. In studying the impacts to leaf margin specifically, the high variability in over sampling periods may better reflect the long term local population sampling of bias (2). Errors in species identification, location, and dates encompassed in bias (1) are assumed to have been improved over time and re-review within the more modern digital herbariums [25].

### Leaf edge margin extraction and analysis

The process of assessing the margins of leaves relied on manual 2D extraction of a boundary around the leaf perimeter. A custom geometry generation program was developed that allowed import of the digital specimen image and scaling of the image to true dimensional size. Once the image was imported and scaled, a piece-wise point hull was manually created around the edge of target leaves. The point hull was created from the bottom of the leaf petiole and extended around the tip of the leaf to the adjacent side of the leaf petiole. Once the point hull was constructed, it is exported for further assessment.

**Fig 4:**
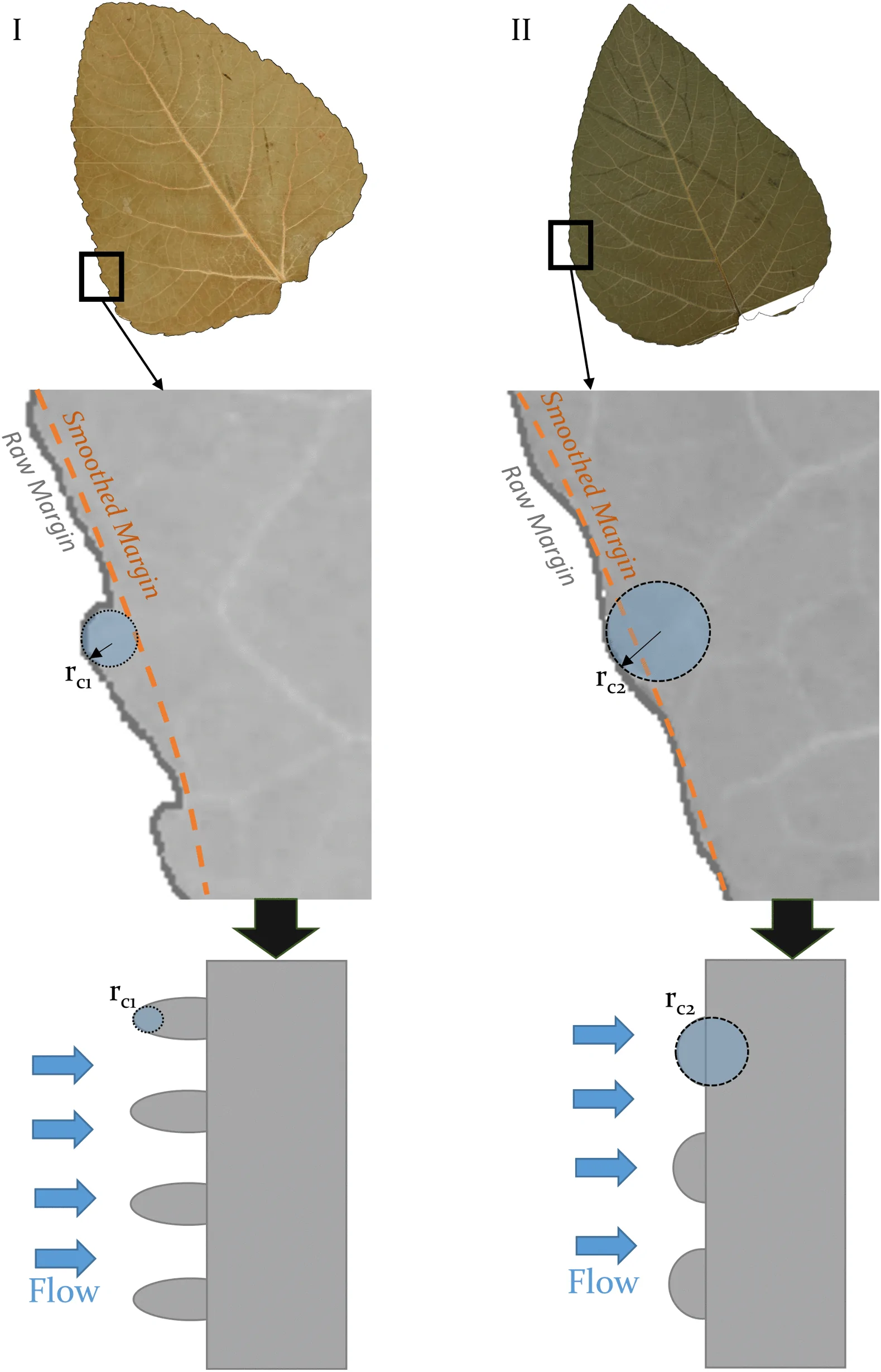
Example of specimen with high edge serration (I) and low edge serration (II). Extracted leaves are shown (top) with inset of edge radii of the leaves (middle), and example of edge undulations for convection structures under cross-flow (bottom). Middle row highlights the raw and smoothed margins used to calculate margin deviation of the specimen.

Prior to leaf parameter measurements, the manually constructed point hulls are resampled to have a uniform 1500 points distributed along the original construction. The up-scaling of the point hulls was utilized to minimize the impacts of sparseness on the hull fitment which could skew edge deviation weighting calculations. Further, a smoothed margin was calculated utilizing a fourth-degree Non-Uniform Rational B-Spline (NURB) spline fitted through the raw margin points [39]. Examples of this smoothing is highlighted in Fig 4. The smoothed margin was leveraged to provide a basis to investigate edge serration differences between specimens.

The leaf margin assessment focused on estimating 2D parameters from the extracted leaf margin. Three primary characteristics of the leaf outline were assessed: aspect ratio (w/l), leaf width (l_c_), and characteristic edge radius. The characteristic edge radius parameter was developed to explicitly tie to applications utilizing perturbations into the flow path of incoming flows such as [40]. Studies into the impact of such undulations have indicated that these structures promote vortical structures that enhance flow stability over a surface [41–43]. Application of similar undulations have been applied to convective surfaces in pursuit of augmenting heat transfer [44]. Edge serration behavior from P. heterophylla is shown in Fig 4 highlighting changes from specimen with deep edge separation to one that is predominantly smooth. In this example, the course edge induces sharper radii nodules that jet from the edge of the leaf. This behavior is contrasted with the smoother example where a larger radii correlates to lower edge roughness. The variation in edge radii may be used to inform cooling structures as illustrated in Fig 4.

The average edge radius was determined by fitting a series of interior circles tangent to the raw leaf outline. An example of this circle-fitting technique is illustrated in Fig 4. Because P. heterophylla exhibits a crenate (scalloped) margin [45], the degree of leaf roughness can be quantified by the size of these fitted radii. In “flatter” or smoother regions of the leaf, the fitted circles are larger, whereas the “creased” or more serrated regions require smaller circles to maintain tangency with the margin’s curvature as observed in Fig 4. To account for variations in overall leaf scale, these radii are non-dimensionalized by dividing them by the average edge deviation between the raw margin and the smoothed envelope, ensuring the resulting metric reflects the specific geometry of the margin rather than the absolute size of the specimen. The characteristic edge radius is then calculated as:


$$\begin{array}{c} r_{c}=\frac{\text{1}}{{N\cdot \delta }_{c}}\cdot \sum r_{e} \end{array}$$ (1)

where

r_c_ – characteristic edge radius

N – number of points a circle as fitted to the leaf outline

r_e_ – circle radius at each margin point

δ_c_ – average deviation of the raw margin edge from the smoothed edge

This parameter allows for an assessment of the extent of edge lobes relative to the width of the leaf. These calculations were then conducted for each of the leaves extracted. Leaves were then segregated based on temperature cluster region. The average values of the three parameters were then compared between the groups to assess differences in populations. To assess the variability of the margin extraction, a repeatability assessment was completed across two samples (one from northern latitudes, one from southern latitudes) with five replicate extractions. Results of this assessment showed that leaf aspect ratio and width variance less than 0.1% of pooled mean values and edge radii estimates varied below 1% of pooled mean values, well below population estimates observed in the study results.

## Results

### Results of cluster-based analysis on leaf specimen segregation

**Fig 5:**
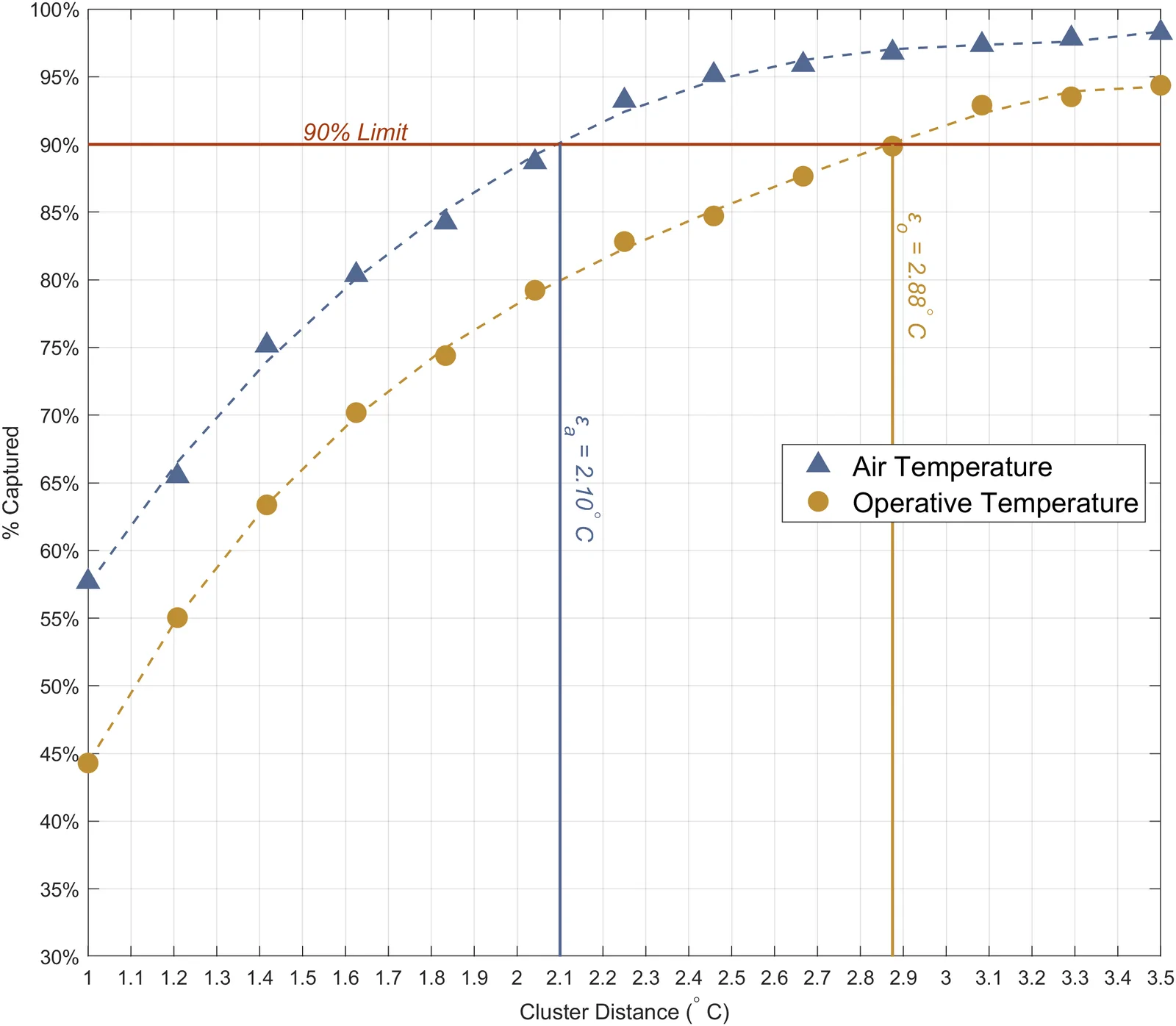
Sensitivity assessment of DBSCAN for both air temperature (blue) and operative temperature (yellow) space. Results are shown for minimum neighbor count of 35.

A sensitivity assessment of optimal search radius (ε) of the cluster analysis was conducted to identify optimal segregation behavior for both air temperature and operative temperature spaces. The study was conducted to identify where 90% of the spatial locations were captured within a cluster. This cut limit was used to identify the critical search radius for the air temperature and operative temperature spaced independently. Results of this sensitivity assessment is shown in Fig 5 illustrating where the 90% captured rate occurred for each temperature space. Multiple iterations of this sensitivity study was conducted across a sweep of minimum neighborhood points to identify when the cluster sizes stabilized (Fig 3 in S2 File). From the results in Fig 5, search radii were found to be ε_a_ = 2.1°C for air temperature space and ε_o_ = 2.88°C for operative temperature space. A k-distance assessment plot assessment was also conducted to ensure that these parameters represent the ‘knee-in-the curve’ for nearest neighbor distances.

**Fig 6:**
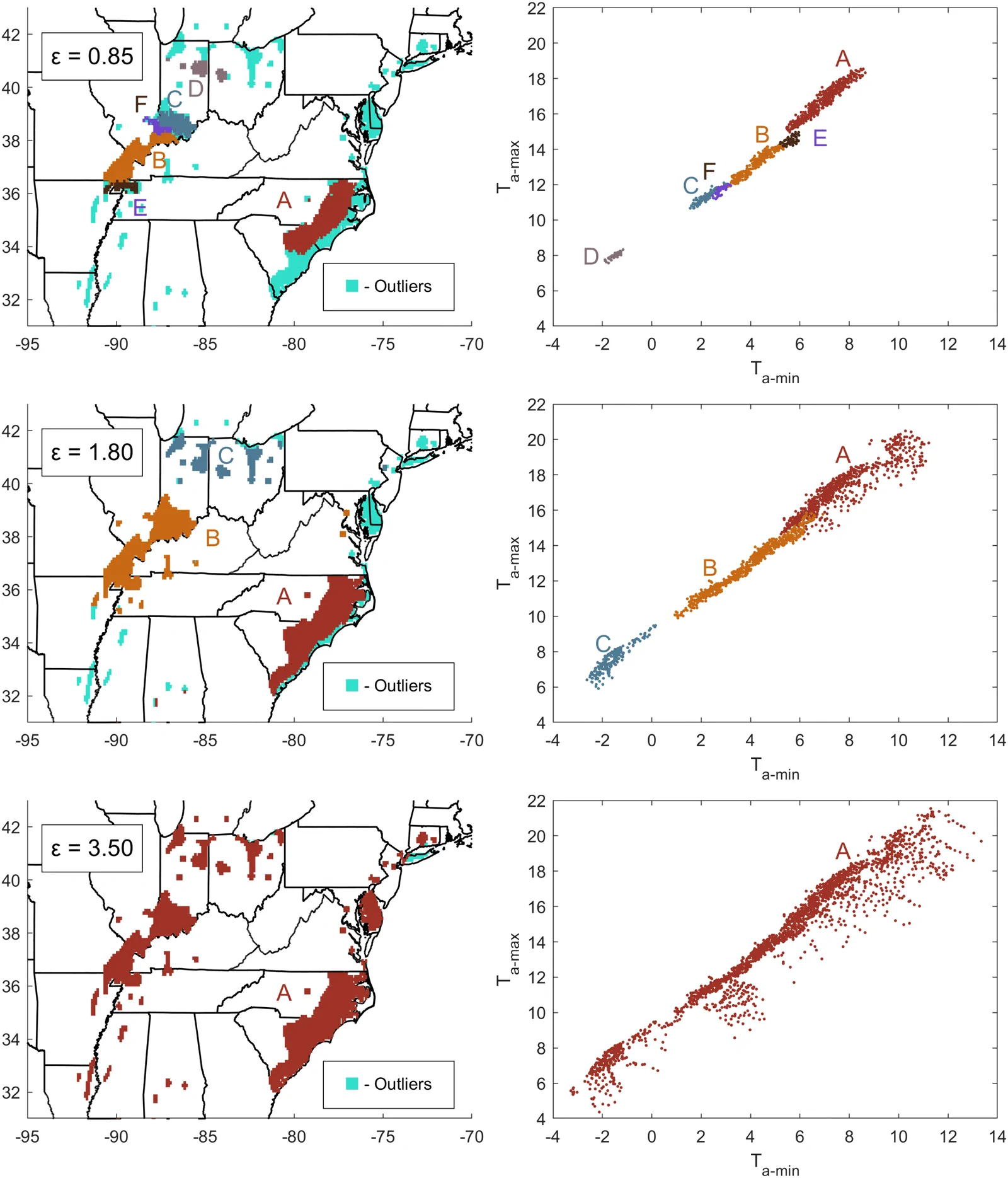
Cluster development results of air temperature space groups. Left plots show emergent clusters overlaid to basal regions. Right plots show corresponding location minimum versus maximum yearly average 2-m air temperatures for captured groupings. The ε = 1.80 reflects the 80% capture of points in air temperature space.

**Fig 7:**
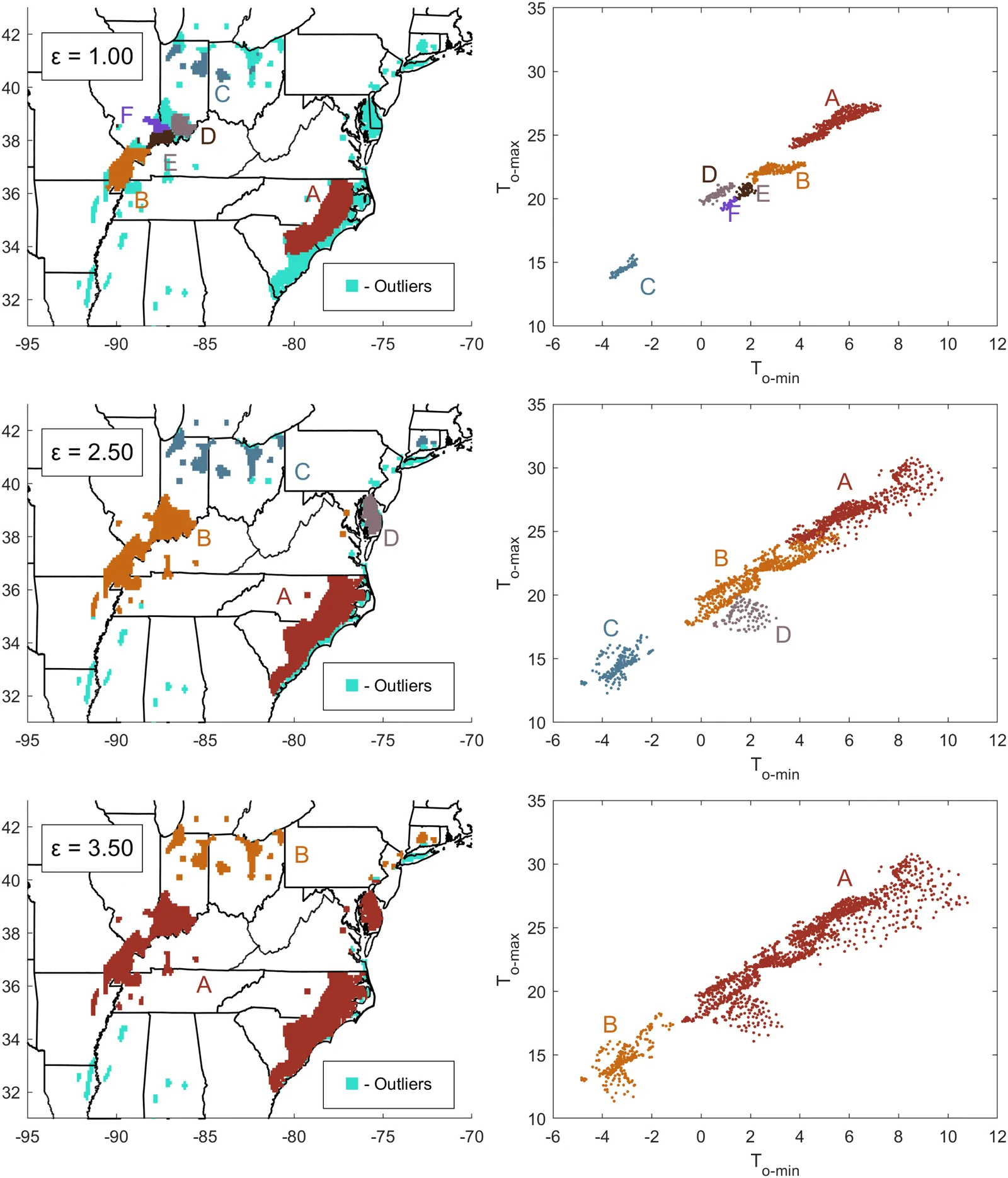
Cluster development results for operative temperature space groups. Left plots show emergent clusters overlaid to basal regions. Right plots show corresponding location minimum versus maximum yearly average operative air temperatures for captured groupings. The ε = 2.50 reflects the 80% capture of points in operative temperature space.

Results for air temperature cluster development as search radius is increased are shown in Fig 6 illustrating the tight constraint of air temperature clustering with small increases in separation magnitude. Here moderate increases to the separation magnitude encompasses a larger accumulation of points across the basal range of the species. In contrast to this, operative temperature space clustering sensitivity results shown in Fig 7 shows this parameter allows for finer gradations of groupings. Middle plots from both Figs 6 and Fig 7 represent the approximately 80% capture of the respective groups.

**Fig 8:**
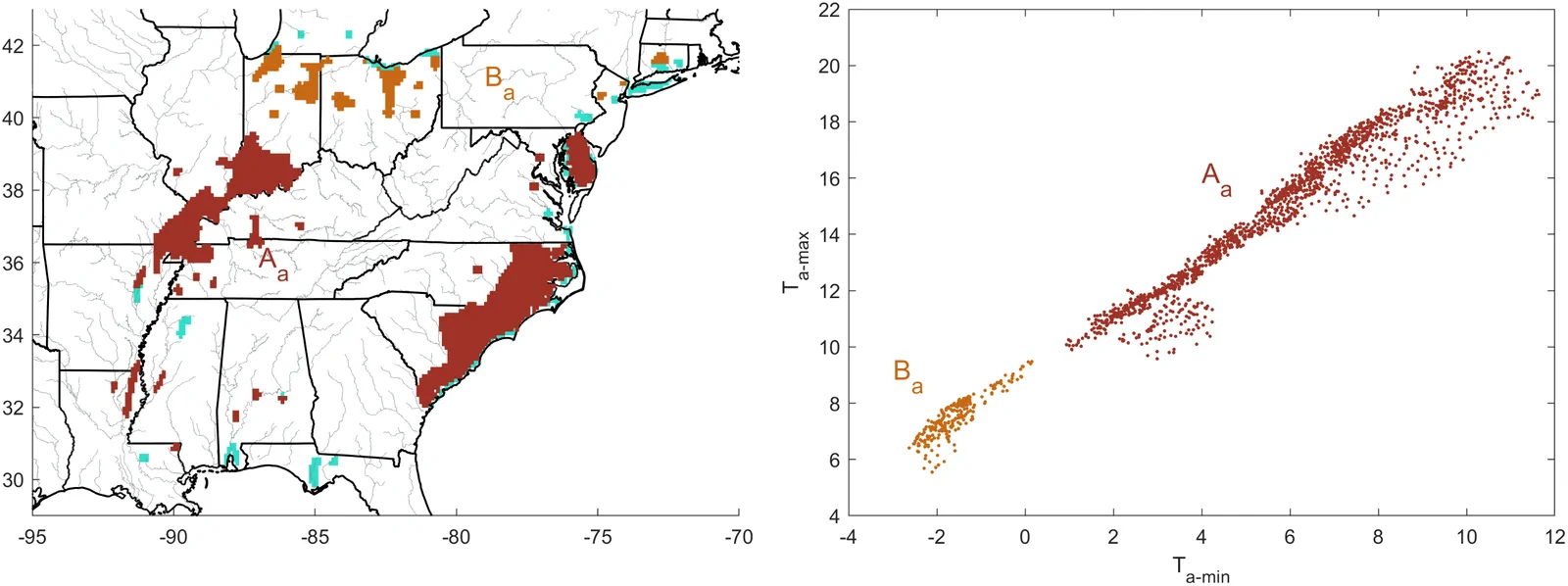
Map of spatial and temperature groupings of P. heterophylla based on maximum, minimum, and average monthly air temperatures. Spatial groupings highlight two large clusters “A_a_” and “B_a_” (left). Location average maximum versus minimum air temperatures with points belonging in “A_a_” colored red and points in group “B_a_” colored in orange (right).

The DBSCAN cluster analysis of the species population basal region was conducted for both air and operative temperature space utilizing a search radius of ε_a_ = 2.1°C and ε_o_ = 2.88°C respectively with a minimum neghborhood point count of 35 to create spatial regions of different heat load the species may be exposed to. Distribution groupings for the clustering-by-air temperature results are shown in Fig 8. The assessment results in two major groupings of the species divided by two large latitudinally distinct regions in the northern portion and southern portions of the species distribution. Grouping *A*_*a*_ is shown to aggregate locations of warmer air temperatures and grouping *B*_*a*_ aggregating cooler distribution locations. Both groups show fairly linear increasing trends between minimum and maximum air temperatures.

**Fig 9:**
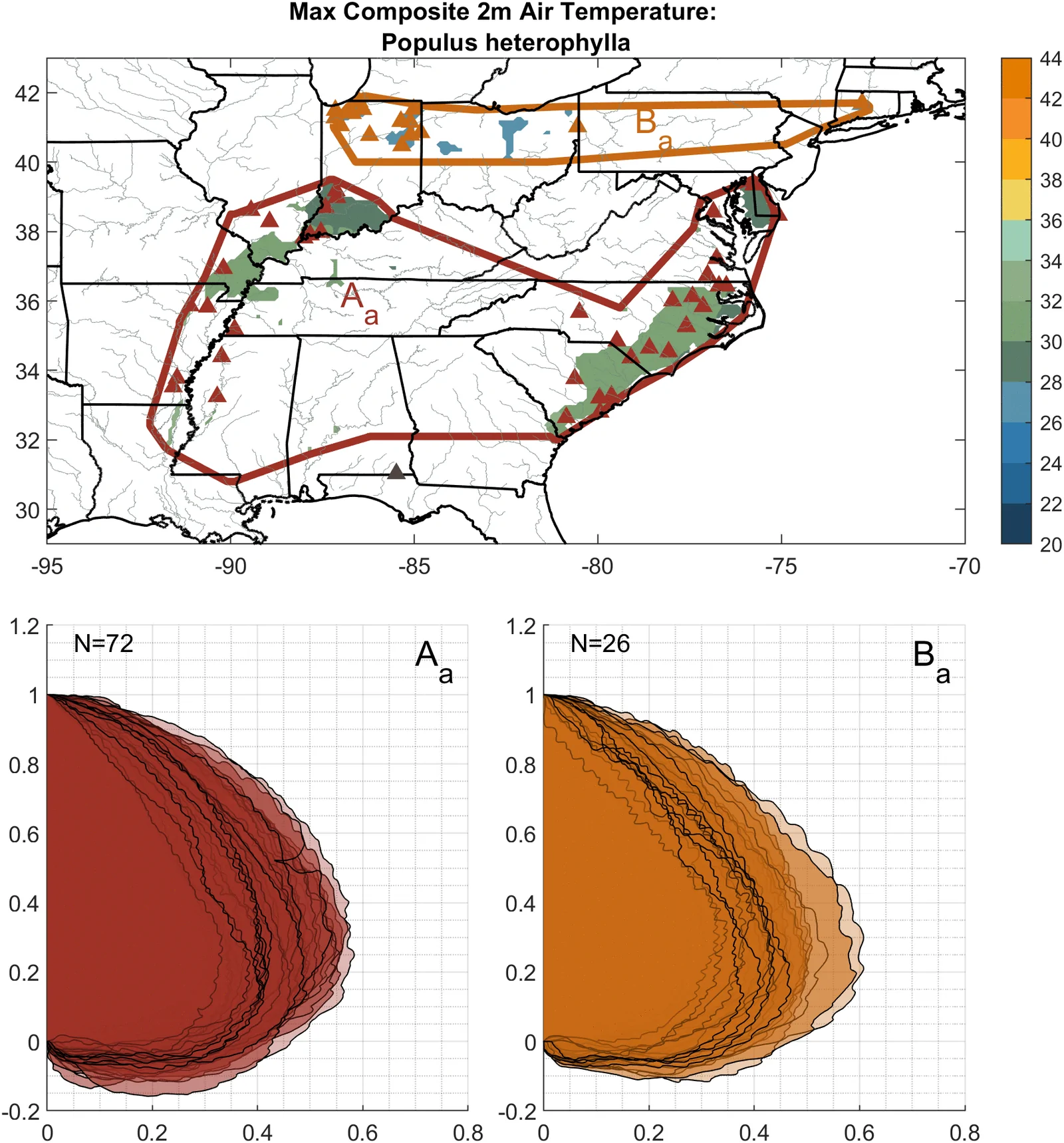
Maximum composite 2m air temperature basal distribution of P. heterophylla and air temperature margin outlines. Temperature-space distribution groupings outlined in red (A_a_) and orange (B_a_) (top chart). Leaf margin specimen locations are highlighted in triangles in species distribution map by cluster color; outliers are plotting in grey triangles. Leaf specimen outline overlays for the temperature-space groupings are plotted and scaled by leaf length (bottom charts denoted “A_a_” and “B_a_”).

Leaf specimens from both regions were assessed on margin outlines and are shown in the bottom graphs of Fig 9. The leaf outlines were non-dimensionalized by leaf length and presented in a one-sided overlay to show trends in margins between groupings. The overlay shows a small clustering of margin aspect ratios of grouping *B*_*a*_ around 0.4 while grouping A_a_ trends shows a larger distribution through the range of 0.4 to 0.6.

**Fig 10:**
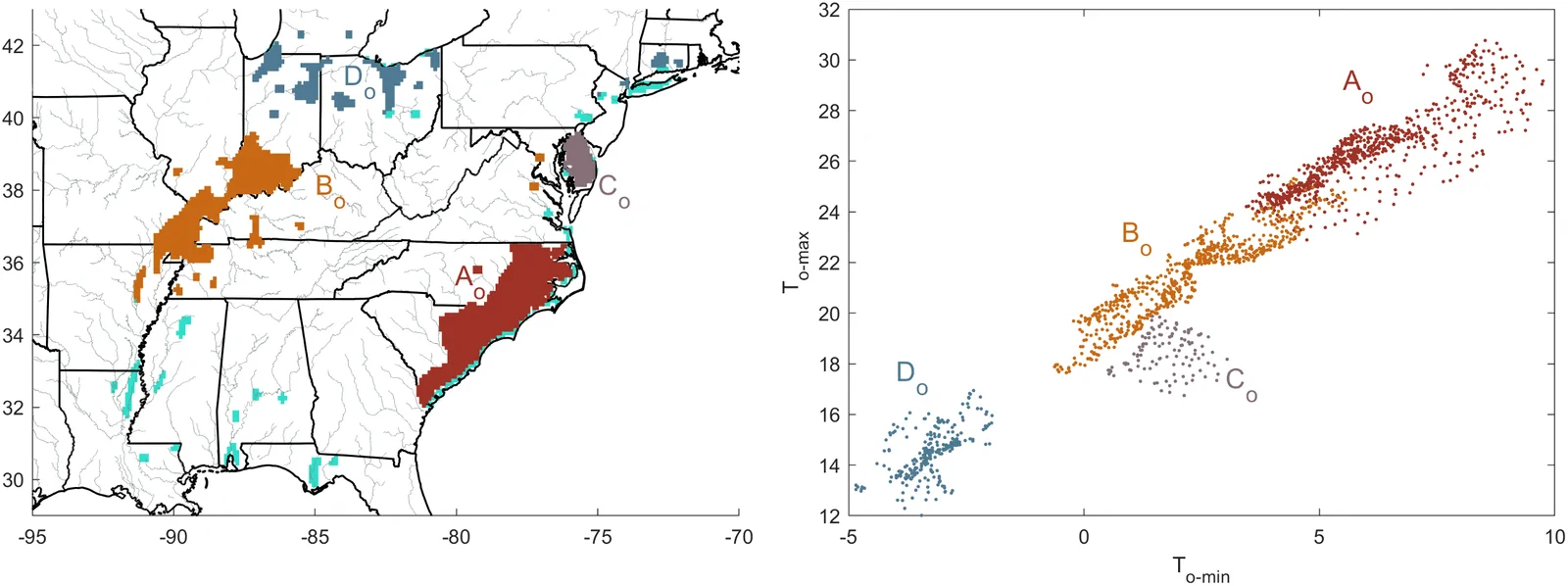
Map of spatial groupings of P. heterophylla based on maximum, minimum, and average monthly operative temperatures. Spatial groupings highlight four large clusters “A_o_”, “B_o_”, “C_o_” and “D_o_” (left). Location average maximum versus minimum air temperatures with points belonging in “A_o_” colored in red, “B_o_” colored in orange, “C_o_” colored in grey, and “D_o_” colored in blue (right).

Results of spatial groupings based on operative temperature space is shown in Fig 10. Here four regions have been identified with only the most northern latitude grouping D_o_ similar from the air temperature grouping results of air grouping B_A_. Lower latitude groups have been split between coastal locations characterized in A_o_ and continental regions of B_o_. It is also important to note these regions are separated by the Appalachian and Blue Ridge mountain ranges. A small distinct cluster, C_o_, has also been identified in the mid-Atlantic states of Maryland and Delaware. This cluster has a minimum yearly temperatures similar to regions in B_o_ but generally have lower maximum temperatures observed in the right hand plot of Fig 10.

**Fig 11:**
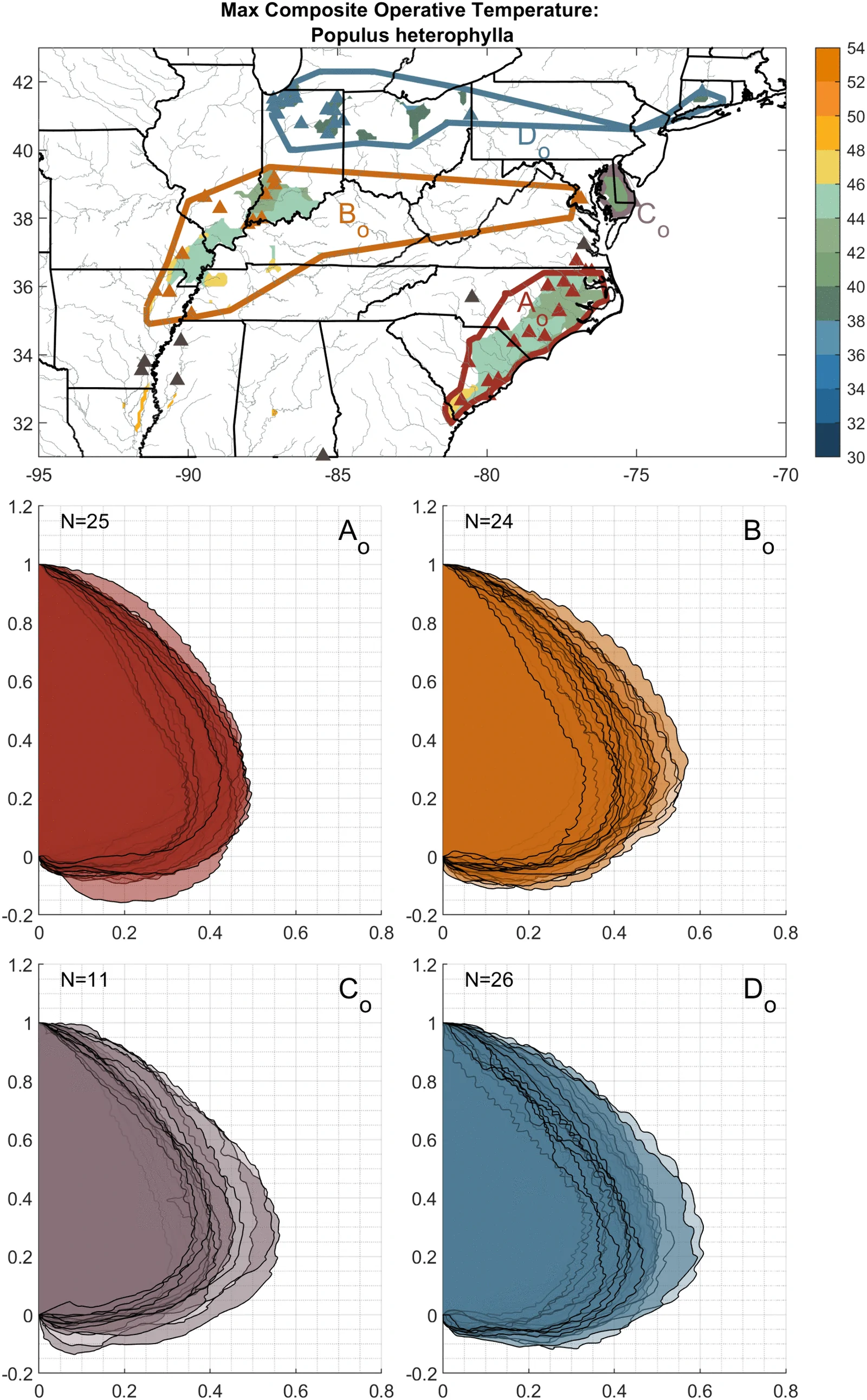
Maximum composite operative temperature basal distribution of P. heterophylla and operative temperature group margin outlines. Temperature-space distribution groupings outlined in red “A_o_”, orange “B_o_”, grey “C_o_”, and blue “D_o_” (top chart). Leaf margin specimen locations are highlighted in triangles in species distribution map by cluster color; outliers are plotting in grey triangles. Leaf specimen outline overlays for the temperature-space groupings are plotted and scaled by leaf length (bottom charts denoted “A_o_”, “B_o_”, “C_o_”, and “D_o_”).

Leaf margin overlays of operative temperature based groups are shown in Fig 11. Qualitative assessments of the overlays indicates that aspect ratio of the warmest cluster (A_o_) is narrower than cooler groups with a majority of the aspect ratios in the range of 0.4 to 0.5. In contrast to this, the other groups have a much larger distribution of aspect ratios ranging from 0.4 to 0.6.

### Results of margin assessments on segregated leaf populations

**Fig 12:**
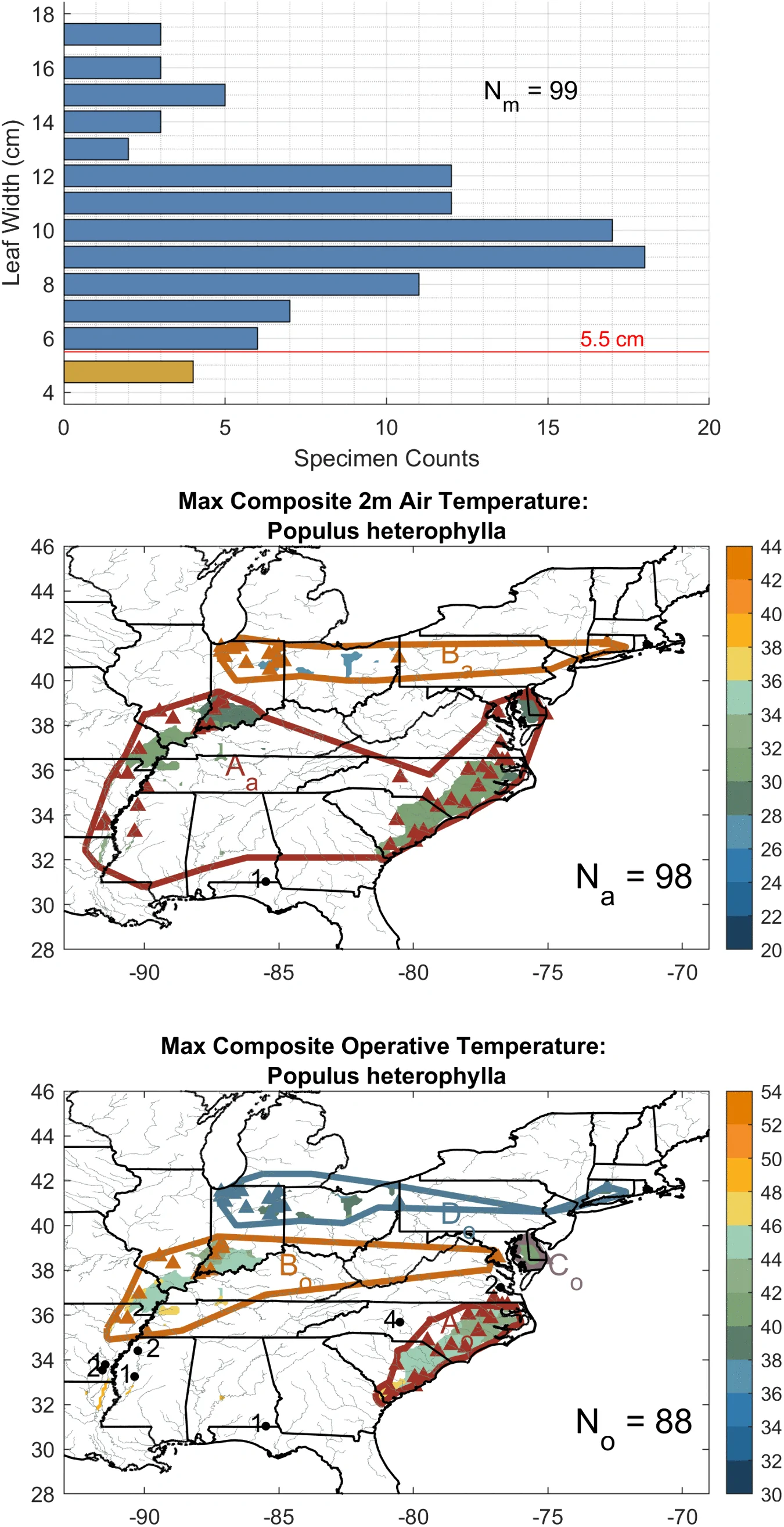
Evolution of specimen population. Top plot shows leaf width histogram of all digitized specimens highlighting those not considered for further processing in the study highlighted in yellow. Middle plot shows capture of specimens by air temperature groups. Bottom plot shows capture of specimens by operative temperature groups. For both middle and lower plots, outliers are plotted with black circles with the number of specimens in the location notated.

Evolution of specimen group size is shown in Fig 12 highlighting the reduction in overall digital specimens to those contained in both the air and operative temperature groups. For the 103 specimens digitized, four were rejected for being smaller than the selection width bounds dropping the overall study number to 99. Further reductions to N_a_ = 98 for the air temperature groups and N_o_ = 88 for the operative temperature groups were observed due to specimen positions residing outside of the defined group regions.

**Fig 13:**
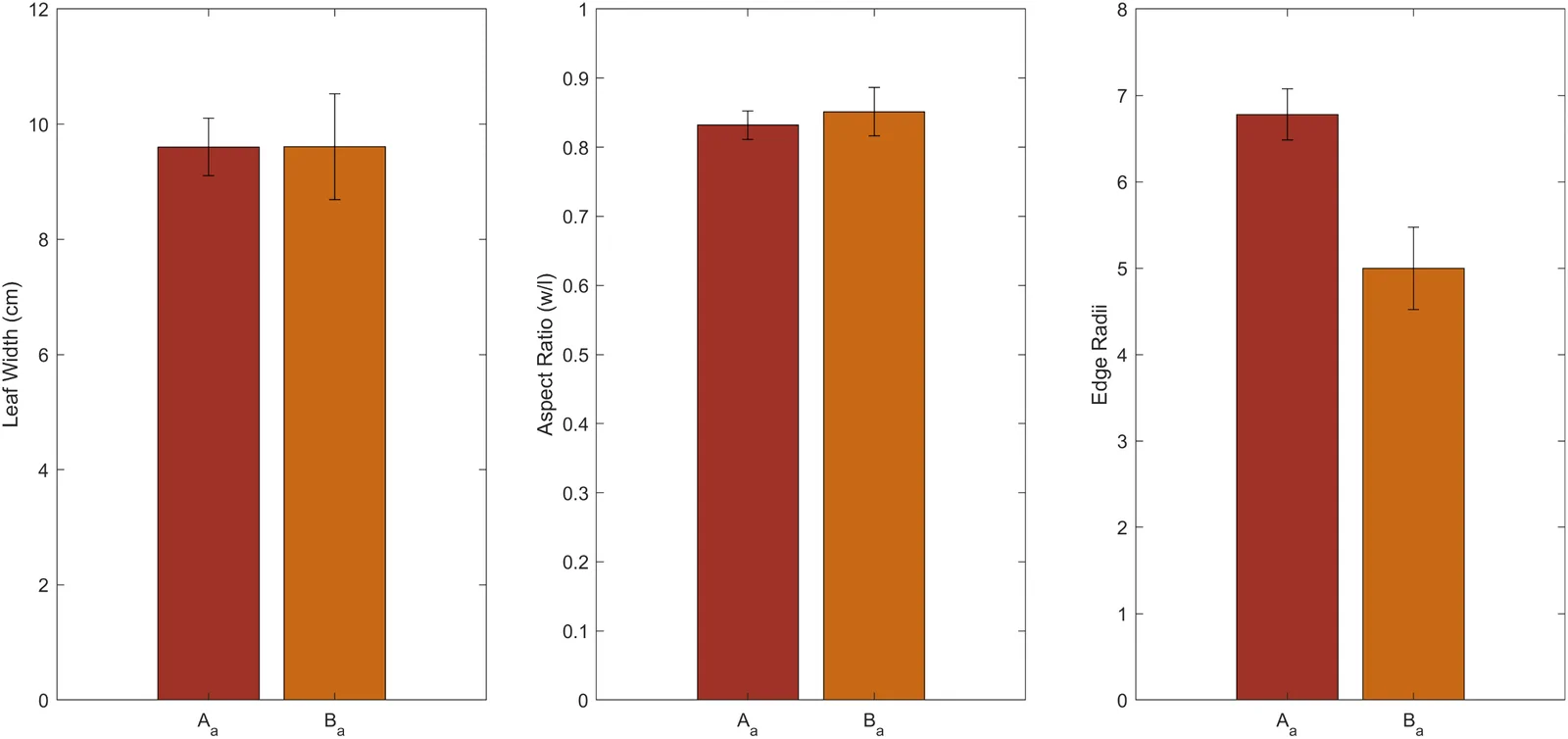
Margin assessment results for ambient temperature groups A_a_ and B_a_. Leaf width (l_c_) shown on left, average aspect ratio in the middle, and non-dimensionalized average edge radii is shown on right. Error bars reflect 95% confidence interval around value means.

Results for the leaf margin assessments of specimen groups based on ambient temperature is shown in Fig 13. As observed in the margin overlays of Fig 9, there is large overlap in aspect ratios between groups. While there is a small increase to aspect ratio for the cooler group B_a_, it is not a significant shift relative to the larger southern grouping A_a_ (independent T-Test, *T*(96) = −0.80*, p* = −0.07). Average leaf width *l*_*c*_ was observed not to follow a normal distribution for group A_a_ (AD = 0.78, n = 72, *p* = 0.04) and a non-parametric test was used to identify the average leaf width between A_a_ and B_a_ to be similar (Wilcoxon signed-rank test, Mdn_a_ = 9.22, Mdn_b_ = 9.32, p > 0.5). Edge radii variation was shown to be significantly different between groups (independent T-Test, *T*(96) = 5.25, *p* < 0.01) with homogeneity of variance between group edge radii values verified per Levene’s Test (*L*(1,96) = 0.18, *p* = 0.67). Group descriptive statistics and goodness of fit parameters for margin parameters are shown in Table 1.

**Table 1:** Leaf margin parameters statistics and A-D Normality Test values for air temperature groupings.

Parameter	Grouping	N	Mean	Std. Deviation	A-D Statistic	A-D *p*-Value
Edge Radii	A_a_	72	6.78	1.50	0.43	0.30
	B_a_	26	5.00	1.42	0.26	0.70
Aspect Ratio	A_a_	72	0.83	0.11	0.59	0.12
	B_a_	26	0.85	0.10	0.72	0.05
Leaf Width (cm)	A_a_	72	9.60	2.53	0.78	0.04
	B_a_	26	9.61	2.75	0.32	0.53

**Fig 14:**
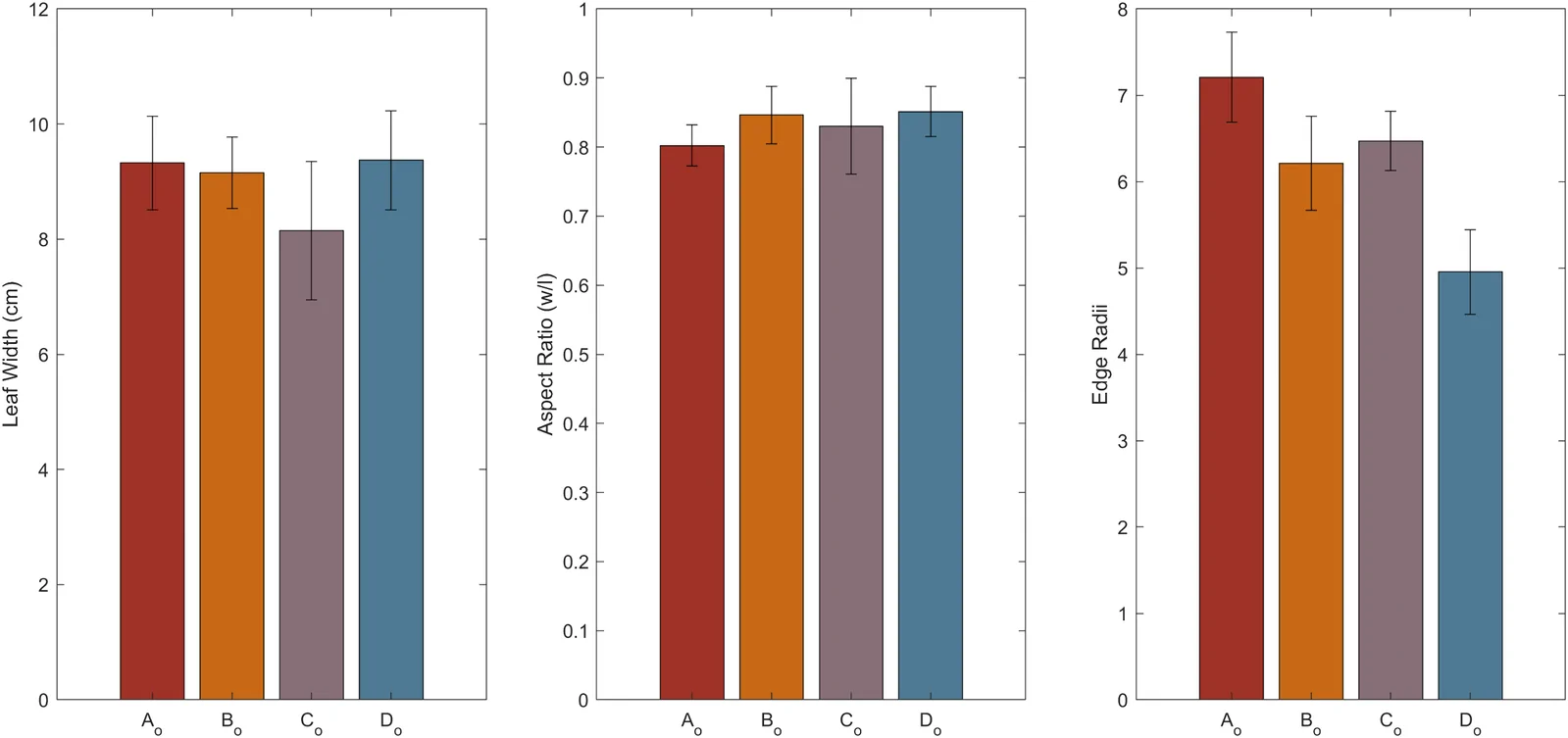
Margin assessment results for operative temperature groups A_o_, B_o_, C_o_, and D_o_. Leaf width (lc) shown on left, average aspect ratio in the middle, and non-dimensionalized average edge radii is shown on right. Error bars reflect 95% confidence interval around value mean.

Leaf margin assessments of specimen groups based on operative temperatures is shown in Fig 14. Leaf aspect ratio shows an increasing trend associated with cooler regions. Leaf widths showed a generally mixed result between groups without a clear trend in direction based on grouping. Differences in aspect ratio were not found to be significant between groups as determined by a one-way ANOVA assessment between means (*F*(3,82) = 1.05, *p* = 0.38). Edge radii variation shows a distinct decrease in radii for the warmest region group A_o_ to group D_o_. The shift in mean edge radii was shown to be statistically significant (one-way ANOVA assessment, *F*(3,82) = 11.00, *p* < 0.05). After verifying homogeneity of variance between group edge radii values (Levene’s Test, *L*(3,82) = 1.61, *p* = 0.19), a post hoc assessment (Tukey HSD) of the groups identified a statistically significant difference only between group A_o_ and D_o_. All data sets were tested for normality (Anderson-Darling Goodness-of-fit test) and were found to not significantly deviate from a normal distribution. Group descriptive statistics and goodness of fit parameters for edge radii for the operative temperature groups are shown in Table 2. This trend in edge radii can be observed by the generally smoother edges observed in the A_o_ margin overlays of Fig 11 and sharper tooth venation of the other groups.

**Table 2:** Leaf margin parameters statistics and A-D Normality Test values for operative temperature groupings.

Parameter	Grouping	N	Mean	Std. Deviation	A-D Statistic	A-D *p*-Value
Edge Radii	A_o_	25	7.23	1.47	0.47	0.23
	B_o_	24	6.21	1.60	0.40	0.34
	C_o_	11	6.47	0.63	0.50	0.17
	D_o_	26	5.00	1.42	0.26	0.70
Aspect Ratio	A_o_	25	0.80	0.08	0.56	0.13
	B_o_	24	0.85	0.12	0.42	0.30
	C_o_	11	0.83	0.13	0.41	0.30
	D_o_	26	0.85	0.10	0.72	0.05
Leaf Width (cm)	A_o_	25	9.62	2.73	0.51	0.18
	B_o_	24	9.15	1.78	0.45	0.26
	C_o_	11	8.15	2.20	0.24	0.74
	D_o_	26	9.61	2.75	0.32	0.53

## Discussion

### Cluster-based population segregation

**Fig 15:**
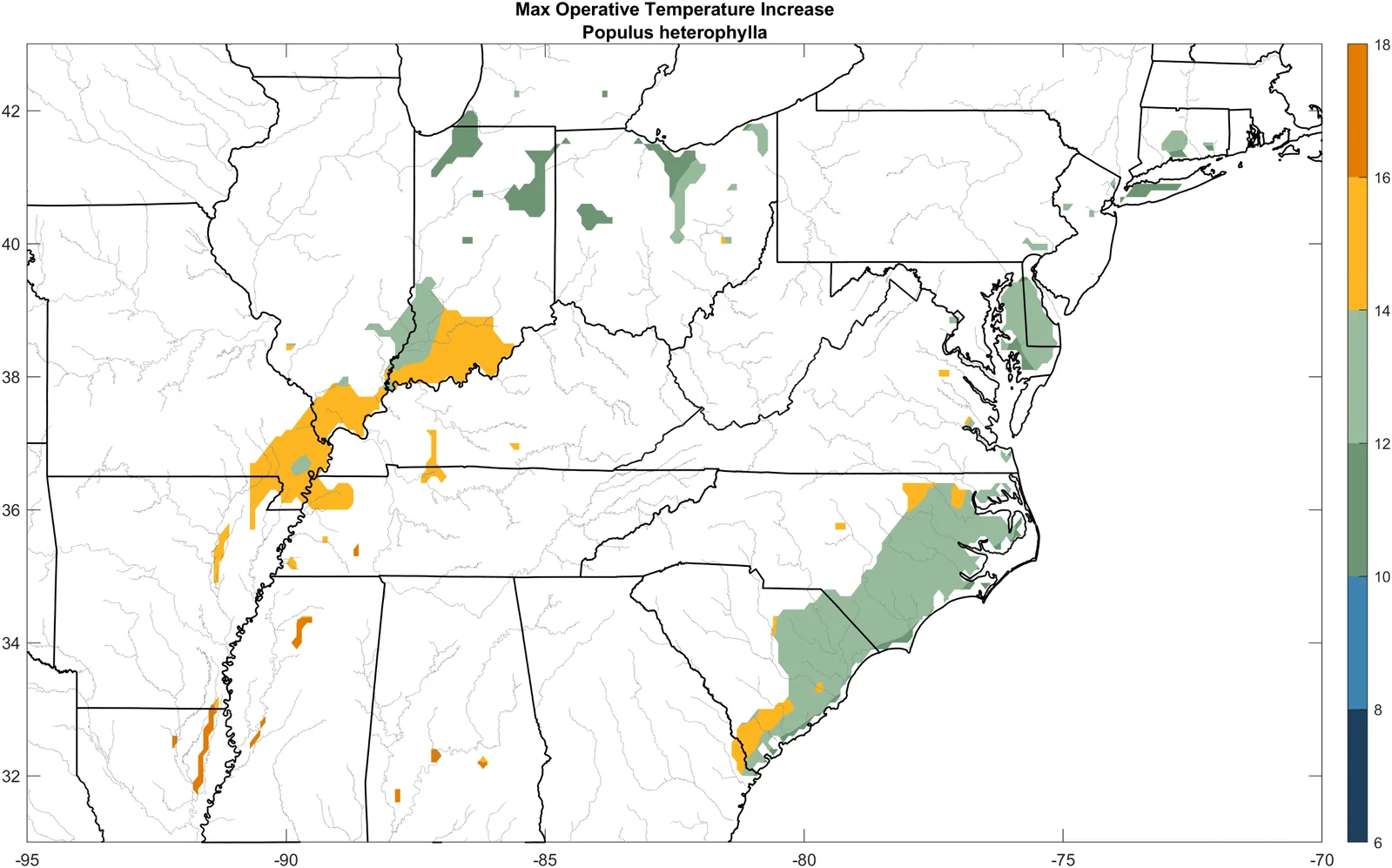
Operative temperature increase over 2m air temperatures for basal locations of Populus heterophyllus. Values are based on location maximum yearly operative temperature difference from current air temperature of maximum occurrence.

Population segregation based in operative temperature space shows a greater differentiation of groups in comparison to population segregation based on ambient air temperatures (Figs 10 and Fig 8). Operative temperature ranges drives differentiation of region environment by a general increase of maximum temperatures and slight amount of cooling due to radiative emission in the overnight hours. As observed in the basal temperature range maps of the study species (left-hand maps in Figs 8 and Fig 10), the maximum operative temperatures the species encounters over the year is on the order of 10–15 degrees Celsius higher than the 2m air temperature illustrated in Fig 15.

**Fig 16:**
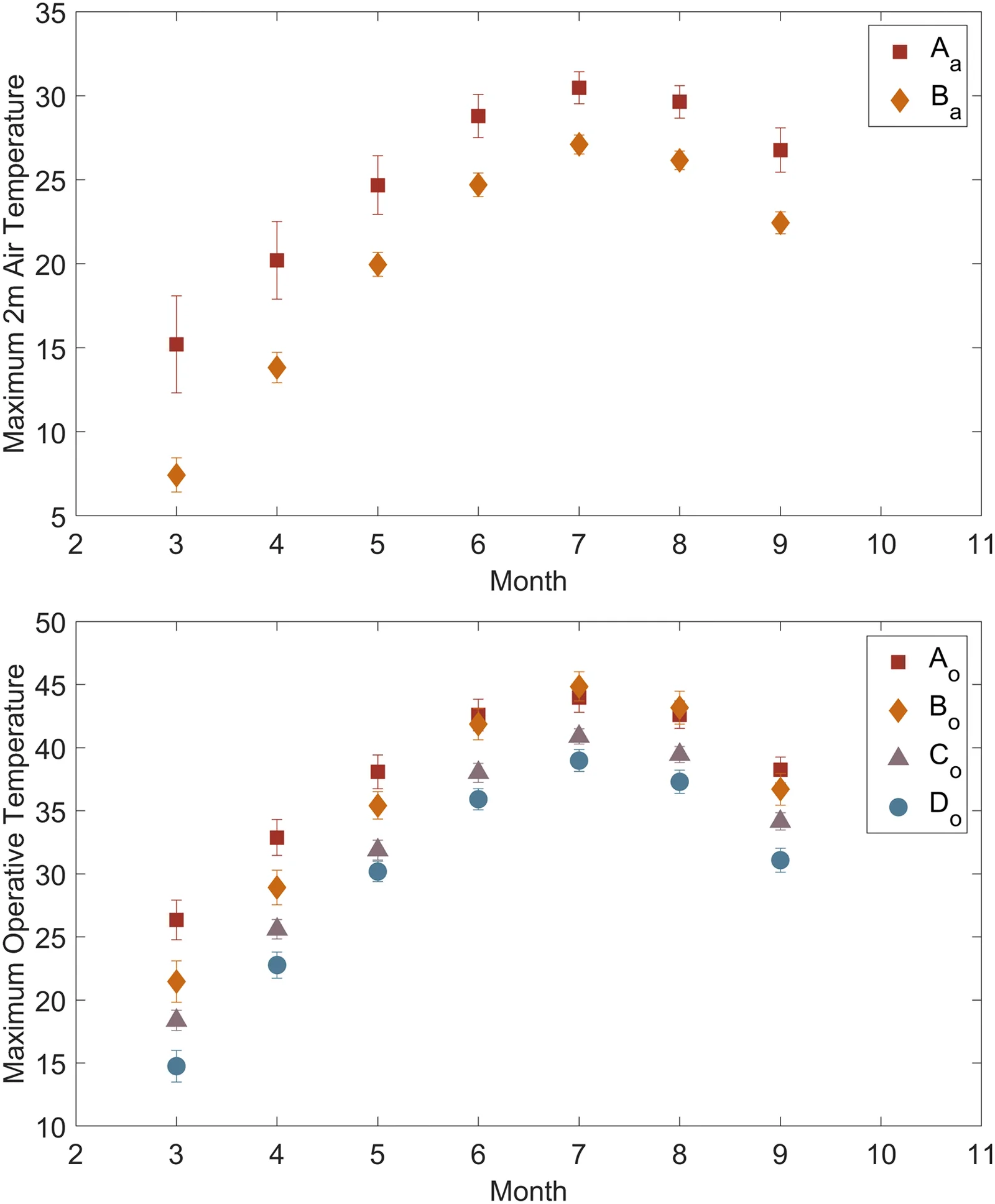
Grouping average monthly maximum 2M Air Temperature (top) and average monthly maximum operative temperature (bottom). Error bars represent standard deviation around value means.

**Fig 17:**
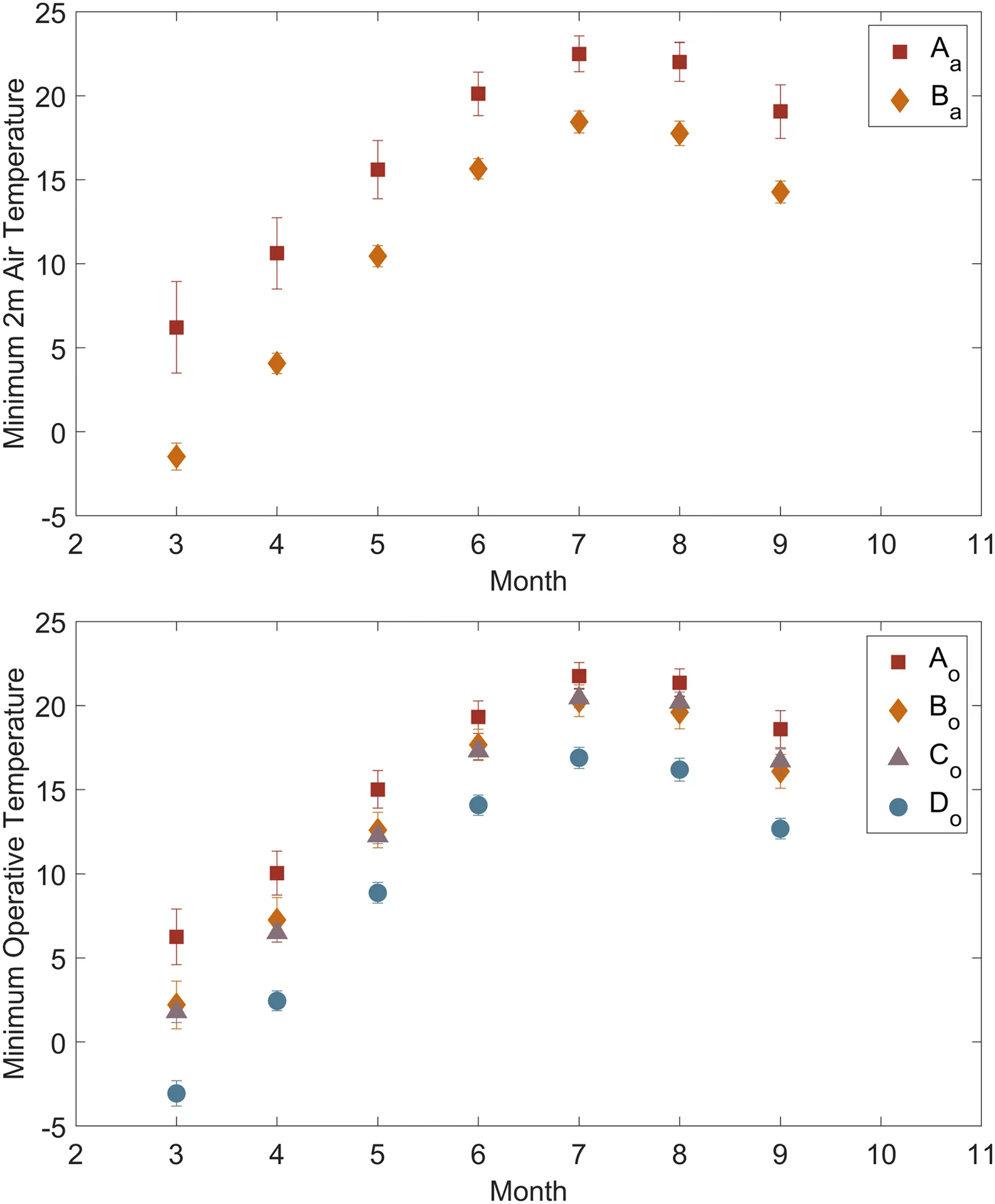
Grouping average monthly minimum 2M Air Temperature (top) and average monthly minimum operative temperature (bottom). Error bars represent standard deviation around value means.

The operative temperature estimates generate an increased thermal parameter range of the species, however it does not reflect a direct step change to all species grid locations. Relying on the solar incidence of the location and relative air speeds that the region has to draw heat away from leaves, the operative temperature provides a more descriptive metric of thermal ecology than air temperature alone. In this manner grid locations are drawn apart in temperature space allowing for an increase in granularity of the environment a species may encounter. Figs 16 and Fig 17 shows the average maximum and minimum temperatures for the spatial groupings of *P. heterophylla*. The elongation of range provides greater monthly granularity in identifying how the temperature-driven spatial groupings behave from month-to-month.

The sensitivity of the clustering methods to nearest neighborhood size and neighbor count is shown in Figs 6 and Fig 7. These parameters were selected to capture 90% of the species grid points (Fig 5 and Supplement Fig 1 in S1 File) while maintaining capture stability (Supplement Fig 2 in S3 File). The evolution of cluster capture highlights a bias in operative temperature space toward including more cool-latitude locations as the capture percentage increases. Specifically, starting at the 80% capture threshold, Fig 7 shows northern locations merging into the cool-weather cluster, whereas Fig 6 demonstrates southern locations being pulled into the warm-weather cluster. These trends support the mechanism that operative temperatures drive location divergence largely due to solar heating impacts.

Separation between all locations is observed to be large in early season months similarly between the air temperature groups and operative temperature groupings. As air temperatures increase into summer, these groups move closer in range but represent distinct separation through month ten. Operative temperature groupings show intermediate groups (B_o_ and C_o_) behavior distinct in monthly trends relative to predominantly warm regions (A_o_) and predominantly cold regions (D_o_). For maximum operative temperatures, these groups trend the space between very cold regions during early and late months while collapsing to hot region temperatures (for B_o_) or sitting between hot and cold regions (for C_o_). For minimum operative temperatures, trends show a collapse of three groups (A_o_, B_o_, and C_o_) into the warmer months while D_o_ remains distinctively lower.

### Margin assessments of *P. heterophylla* samples

One-hundred-and-three specimens from digital herbarium archives were digitized and used within this study. The specimens were taken from digital collections, outlines of selected leaves were extracted, and estimates of leaf width, aspect ratio, and non-dimensionalized edge radii were calculated. The one-hundred-and-three were first culled by leaf width standards and further reductions in study population realized after grouping per air temperature spatial regions (shown in Fig 8) and again for operative temperature spatial regions (shown in Fig 10). The evolution of the study populations is illustrated in Fig 12. The average values between groups were then compared and statistical comparisons performed to identify significance in the observed means.

As observed in Fig 12, the original specimen set was culled from the 103 digitized specimens to 98 specimens used in the air temperature assessments and 88 used for the operative temperature assessments. This drawing down was based on width range criteria intended to ensure equal maturity levels across the variable growing seasons and further condensing based on temperature group spatial inclusions. To ensure results of air temperature group margin assessments were not a product of differing population size in relationship to the operative temperature groups, a secondary assessment of air temperature groups was conducted using only specimens that were utilized in the operative temperature group analysis (see supplement 3 Figures 5 and 6 in S3 File). Results of this subset assessment remained consistent with the full 98 specimen set supporting consistency of analysis between population subsets.

Both air temperature driven and operative temperature groups showed increases to leaf aspect ratio and widths with groupings associated with cooler climates. Both groupings also showed a decrease in edge radii with cooler climates indicating higher edge dissection and teething. This is consistent with other studies observations of greater leaf teeth dissection along latitudinal and air temperature gradients [15,19,46]. The greater temperature range provided by spatial group construction using operative temperatures creates two additional groups beyond what the air temperature case has generated. These are generated in a mid-latitude regions and are separated between a largely continental environment (group B_o_) and a distinct coastal location (group C_o_). As observed in Fig 16 and Fig 17, these two groupings share similar cool month behaviors between A_o_ and D_o_ groups however they separate as group B_o_ operative temperatures match those of the hot climate A_o_ and C_o_ stays between the cool region group D_o_ and the warm climate groups.

The magnitudes of variation observed in the leaf edge radii trends represent small variations to the leaf profile relative to the magnitude of leaf widths observed. For both grouping sets the results point to trends in morphological behavior relative to environmental drivers. Air driven groups A_a_ and B_a_ average temperatures are observed to drive to a similar peak during summer months similar to operative temperature groups A_o_ and D_o_ both associated with a reduction in edge radii from warm regions to cold regions. This edge radii reduction is observed in the emergent mid-latitude groups B_o_ and C_o_, however they do not present statistically significant differences to A_o_. This statistical overlap may be due to seasonal or local environmental impacts that provide a generally coincident environment to over-lapping latitudes of group A_o_. Both group B_o_ and C_o_ are observed to have similar warm weather operative peaks as group A_o_ but are markedly colder during early growth months 3–5. These months may provide the environmental stimuli to drive the trend in decreased edge radii observed in Fig 14. Further study into cooler weather dynamics would be warranted to identify how margin serration would impact leaf energy transport. These studies would coincide with the hypothesis that leaf margin serration is aligned to early season growth dynamics [19] which contends that roughened leaf margins drive higher diffusive transfer at the edge of the leaves aiding in early season transport [47].

Future work to leverage these findings are to create appropriate analogues of the morphological change of leaf edge radii applied to a problem of interest. With the current results indicating the morphology tracks with adaption to augment edge diffusion, situations in need of increasing ejection efficiency such as [40,48] may be an applicable design space this would be suited for. Finally, future studies of the operative temperature paradigm would work to incorporate two-phase heat transfer considerations based on local water availability through the year.

### Limitations of the parameter

The operative temperature metric developed in [5] is intended to serve as an abiotic climate parameter that integrates the impacts of environmental radiative and convective heat loads experienced by an organism. The parameter is not intended to be interpreted as a direct modeling estimate of a species thermal relationship to the environment or heat transfer response, rather it strives to provide an estimate of the heat load severity of the meso-scale climate. This parameter provides a baseline context to investigate species differentiation along a thermal cline while accounting for environmental heat flux in a singular value. By consolidating multi-mode environmental heat fluxes into a single index, this parameter offers a framework for identifying candidate morphological features associated with regional thermal variation, without assuming specific underlying physiological mechanisms.

The operative temperature utilized does not incorporate transpiration cooling mechanisms that are a major method of temperature control of plants [49]. Incorporation of transpiration cooling would necessitate assumptions of physiological response that would impact the general applicability of the parameter. While the parameter assumes a base size for convective and radiative balance with the environment, physiological features such as leaf mass or stomata response rates would shape the operative temperature to implicitly reflect the environment from the perspective of species with these types of features. The parameter is intended to be used as an environmental reference that would indicate where further taxa-specific research would be valuable. Further work is needed to derive a hydrological parameter than can be leveraged that would not need this physiological assumption and skew results toward species with pre-determined behaviors.

A further consideration of the parameter’s sensitivity to leaf width (l_c_) relative to cluster assessments must also be made. As illustrated in Fig 1, modest increases in the l_c_ leads to moderate increases to operative temperatures relative to the cluster separation parameters (ε_a_ and ε_o_) developed from the 90% capture criteria in Fig 5. Generally, an increase to l_c_ will lead to increases in maximum temperatures due to the increase in energy flux through ambient surroundings. The currently used 3 cm l_c_ has shown to drive up to 18C of yearly maximum temperature difference over location air temperatures as shown in Fig 15. This scaling of temperature space is a strong driver of the magnitudes of ε_a_ and ε_o_ and subsequently the resolution of clusters of interest. Further studies involving species with smaller and larger average leaf sizes would be of interest to better understand the relationship of the l_c_ assumption, ε_o_ magnitude, and cluster resolution.

## Conclusions

Expanding upon the heat transfer-oriented screening methodology of Slavens & Shabgard [5], this study demonstrates that operative temperature-based mapping provides finer environmental resolution for *P. heterophylla* than ambient air temperature alone, resolving four distinct clusters compared to two under the selected clustering settings. While ambient air temperature clustering obscured subtle regional variations, the operative temperature metric revealed distinct sub-populations with morphological shifts. Specifically observed differentiated trends in leaf edge radii across operative temperature-based clusters bolster validity of this parameter as a more granular metric for candidate morphology changes across different thermal systems. This differentiation was most pronounced between the extreme clusters (A_o_ and D_o_), whereas intermediate groups (B_o_ and C_o_) showed less consistent separation. These observed regional patterns align with the hypothesis that cool-season conditions, rather than summer maximums alone, may influence leaf margin variations. Further studies are needed to evaluate whether these reduced edge radii yield functional heat-transfer benefits that could inform bio-inspired engineering designs.

## Supporting information

S1 File Specimen Data. Leaf specimen geometry information, margin statistics, repeatability study results, and margin correlation information.
(XLSX)

S2 File Specimen References. Table of References for Leaf Specimen Sources.
(DOCX)

S3 File Additional Study Results Figures. Clustering stability results Figures 1–4 and results of sub-population assessments for air space in Figures 5 & 6 and Tables 1 & 2.
(DOCX)
