# Supplementary material for: Beyond air temperature: An examination of leveraging operative temperature space for the discovery of bioinspired thermal design features
Source: PLoS One. 2026 Sep 16;21(9):e0358463. doi: 10.1371/journal.pone.0358463 (PMC13580958; doi:10.1371/journal.pone.0358463)
Supplement: Supplementary file 1 [file pone.0358463.s002.docx]

Table of References for Leaf Specimen Sources for Manuscript “Beyond air temperature: An examination of leveraging operative temperature space for the discovery of bioinspired thermal design features”

U.S.A., Delaware: SW along Philadelphia, Baltimore and Washington, 24 May 1909, Long,
<https://portal.torcherbaria.org/portal/collections/individual/index.php?occid=21088600>
<https://portal.torcherbaria.org/portal/collections/individual/index.php?occid=30064807>
<https://portal.torcherbaria.org/portal/collections/individual/index.php?occid=16523690>
<https://portal.torcherbaria.org/portal/collections/individual/index.php?occid=21088598>

U.S.A., Indiana: About 3 miles north of La Porte on the Springville pike, La Porta, 13 August 1911, Deam 39394,
<https://portal.torcherbaria.org/portal/collections/individual/index.php?occid=12537628>

U.S.A., Indiana: about .75 mi north of Bushrod, Greene, 26 May 1912, Deam 39933,
<https://portal.torcherbaria.org/portal/collections/individual/index.php?occid=12537651>

U.S.A., Indiana: about 10 mi. northwest of Knox, Starke, 01 June 1916, Deam 39688, <https://portal.torcherbaria.org/portal/collections/individual/index.php?occid=12537757>

U.S.A., Indiana: 1.25 mi. east of Palmyra, Harrison, 24 June 1916, Deam 39957, <https://portal.torcherbaria.org/portal/collections/individual/index.php?occid=12537650>

U.S.A., Indiana: In Sec. 3 about 0.75 mi. northeast of Summit, DeKalb, 14 June 1928, Deam 39911, <https://portal.torcherbaria.org/portal/collections/individual/index.php?occid=12537656>

U.S.A., Delaware: ca. 2 mi. NW of Fenwick Light, Sussex, 30 May 1939, Schmid PH00572313, <https://portal.torcherbaria.org/portal/collections/individual/index.php?occid=21088607>

U.S.A., Indiana: Vanderburgh, Evansville, 04 June 1941, Zeiner 43622, <https://portal.torcherbaria.org/portal/collections/individual/index.php?occid=12537752>

U.S.A., North Carolina: Lenoir, Kinston, 15 June 1947, Whitford NCSC00014518, <https://portal.torcherbaria.org/portal/collections/individual/index.php?occid=8725323>

U.S.A., Tennessee: Between Raleigh and Pearly, Shelby, 29 June 1948, Fairchild NCSC00014544, <https://portal.torcherbaria.org/portal/collections/individual/index.php?occid=8725349>

U.S.A., North Carolina: 1 mi. below bridge near Rt. 95, about 5 mi. n. e. of Rocky Mount, Edgecombe, 17 June 1947, Fox NCSC00014511, <https://portal.torcherbaria.org/portal/collections/individual/index.php?occid=8725316>

U.S.A., North Carolina: Low woods south of Lauringburg near S. C. line, Scotland, 20 May 1961, Hardin NCSC00014482, <https://portal.torcherbaria.org/portal/collections/individual/index.php?occid=8725287>

U.S.A., North Carolina: 0.3 mi E jct NC 53 & US 421 on NC 53, Pender, 13 June 1969, Case SC002771, <https://portal.torcherbaria.org/portal/collections/individual/index.php?occid=8192174>

U.S.A., Missouri: Mingo Wildlife Refuge, Wayne, 01 May 1971, Brooks 270194, <https://portal.torcherbaria.org/portal/collections/individual/index.php?occid=22474855>

U.S.A., Arkansas, Clay, 15 June 1975, Richards CAU00004324, <https://portal.torcherbaria.org/portal/collections/individual/index.php?occid=19750023>

U.S.A., North Carolina: parallel US 158, 2 miles E of escarpment, Gates, 28 April 1977, Pitchford 5051,
<https://portal.torcherbaria.org/portal/collections/individual/index.php?occid=22805153>

U.S.A., North Carolina: Salisbury Nature Study Area, Salisbury, 6 June 1975 Baranski UNCC_50475, <https://portal.torcherbaria.org/portal/collections/individual/index.php?occid=30412956>
<https://portal.torcherbaria.org/portal/collections/individual/index.php?occid=30412953>

U.S.A., Indiana: Cypress Slough 4 miles SE of Mt. Vernon, Posey, 26 June 1980, Ebinger EIU014676, <https://portal.torcherbaria.org/portal/collections/individual/index.php?occid=8700763>

U.S.A., South Carolina: Richland, Hopkins, 17 June 2008, Zomlefer GA265610, <https://portal.torcherbaria.org/portal/collections/individual/index.php?occid=20845642>

U.S.A., Indiana: Chapine Woods, Whitley, 13 June 2023, Namestnik 166801, <https://portal.torcherbaria.org/portal/collections/individual/index.php?occid=33315411>

U.S.A., North Carolina: Collected 5 mi NW of Fair Bluff, Robeson, 31 July 1969, Oxendine PEMB0003861, <https://portal.torcherbaria.org/portal/collections/individual/index.php?occid=16885910>

U.S.A., Illinois: about 3 miles SW of Mt. Vernon, Jefferson, 19 July 1976, Ebinger EIU014464, <https://portal.torcherbaria.org/portal/collections/individual/index.php?occid=4905924>

U.S.A., Mississippi: Sidon, Leflore, 01 July 1996, MacDonald MISS0017180, <https://portal.torcherbaria.org/portal/collections/individual/index.php?occid=5884332>

U.S.A., South Carolina: McPhersonville Rd. along Buckfield Plantation, Beaufort, 04 July 2006, Payne CLEMS0065737, <https://portal.torcherbaria.org/portal/collections/individual/index.php?occid=19807198>

U.S.A., Indiana: 1 mi. west of the Ohio State Line, Adams, 31 August 1920, Deam 39860, <https://portal.torcherbaria.org/portal/collections/individual/index.php?occid=12537665>

U.S.A., Arkansas: Edge of swamp along Highway 65, Desha, 15 August 1932, Davis UARK055137, <https://portal.torcherbaria.org/portal/collections/individual/index.php?occid=13241361>

U.S.A., Indiana, Little Cypress Swamp, Knox, 05 August 1932, Cain NCSC00014604, <https://portal.torcherbaria.org/portal/collections/individual/index.php?occid=8725409>

U.S.A., Massachusetts: shore of Turkey Hill Pond, Paxton, 22 August 1943, Gates CONN00189924, <https://websites.rbge.org.uk/cgi-bin/nph-readbtree.pl/allspecs?&ETI=CONN00189924>

U.S.A., North Carolina: N. bank of Cape Fear R, Bladen, 28 August 1948, Fox NCSC00014513, <https://portal.torcherbaria.org/portal/collections/individual/index.php?occid=8725318>

U.S.A., Mississippi: Flag Lake swamp 2.8 mi SW of Sledge, Quitman, 14 August 1967, Pullen 22859, <https://portal.torcherbaria.org/portal/collections/individual/index.php?occid=5884338>

U.S.A., Illinois: 4 miles W of Carlyle, Clinton, 13 August 1984, Ebinger EIU014460, <https://portal.torcherbaria.org/portal/collections/individual/index.php?occid=8660047>

U.S.A., Indiana: in the Markley woods, St. Joseph, 14 September 1929, Deam 39977, <https://portal.torcherbaria.org/portal/collections/individual/index.php?occid=12537755>

U.S.A., Arkansas: Cleveland Co., Rye, 30 August 1936, Demaree 2470512, <https://portal.torcherbaria.org/portal/collections/individual/index.php?occid=29141907>

U.S.A., Massachusetts: About 0.4 miles north of intersection of Lithia Springs Road and Elmer Brook ,South Hadley, 1 September 1999, Searcy CONN00164231, <https://websites.rbge.org.uk/cgi-bin/nph-readbtree.pl/allspecs?&ETI=CONN00164231>

U.S.A., Arkansas: Jackson, 2.7 miles north of Swifton, 04 October 1991, Richards STAR028749, <https://portal.torcherbaria.org/portal/collections/individual/index.php?occid=6694374>

U.S.A., Indiana: Timothy Road south of I-80/90, St. Joseph, 21 October 2015, Namestnik 0087816MOR, <https://portal.torcherbaria.org/portal/collections/individual/index.php?occid=13246095>

U.S.A., Indiana: In Sec. 3 about 0.75 mi. northeast of Summit, DeKalb, 14 June 1928, Deam 39911, <https://portal.torcherbaria.org/portal/collections/individual/index.php?occid=12537656>

USA; Connecticut; Hartford County; Southington; swamp in front of High Road, Charles H. Bissell, 1902-07-15, CBS.018716
<https://collections.peabody.yale.edu/search/Record/CBS.018716>

USA; Connecticut; New Haven County; North Guilford; swamp intersection Route 77 & Route 80, John Souther, 1996-10-07, CBS number 18719; lot count 1
<https://collections.peabody.yale.edu/search/Record/CBS.018719>

USA; Connecticut; Middlesex County; Middletown, Charles H. Bissell, 1903-09-02,	CBS number 18722; lot count 1
<https://collections.peabody.yale.edu/search/Record/CBS.018722>

USA; Connecticut; New Haven County; Southbury, Edgar B. Harger, 1908-08-29, CBS number 18724; lot count 1
<https://collections.peabody.yale.edu/search/Record/CBS.018724>

USA; Connecticut; New Haven County; Southbury; Rattlesnake Hill, Leslie J. Mehrhoff, 1984-08-30, CBS number 18725; lot count 1
<https://collections.peabody.yale.edu/search/Record/CBS.018725>

USA; Connecticut; Hartford County; Southington, Charles A. Weatherby, 1924-06-07, YU number 53196; lot count 1
<https://collections.peabody.yale.edu/search/Record/YU.053196>

USA; Connecticut; Hartford County; Southington, Charles A. Weatherby, 1924-06-07, YU number 53197; lot count 1
<https://collections.peabody.yale.edu/search/Record/YU.053197>

USA, Indiana, Clay County; Muir Lake just W of bridge on Rd 59, ca 1 mi E of Howesville, Ray C. Friesner, KU 270198,
<https://biodiversity.ku.edu/botany/vascular-plant-collection>

USA, North Carolina, Lenoir County; Kinston, Larry A. Whitford, 1947-06-15, KU 270199
<https://biodiversity.ku.edu/botany/vascular-plant-collection>

USA, North Carolina, Pitt, Along Green Mill Run, ca 0.1 mi N of 14th Street, Greenville, Mary C Helms; George H. Harvey, 1969-05-16, KU 270195
<https://biodiversity.ku.edu/botany/vascular-plant-collection>

USA, Indiana, Wells, in Sec. 19 of Lancaster Township, Charles C. Deam, 1903-05-24, IND-0070604
<https://portal.torcherbaria.org/portal/collections/individual/index.php?occid=12537745>

United States, Delaware, New Castle, ca. 1 mi. S of Vandyke, Bayard Long, 1940-07-05, PH00572310
<https://midatlanticherbaria.org/portal/collections/individual/index.php?occid=21088604>

USA, North Carolina, Martin, Conoho Creek, Rt. 125. Swamp forest. Martin County, North Carolina, David M. DuMond, 1966-09-30, NCSC00014528
<https://portal.torcherbaria.org/portal/collections/individual/index.php?occid=8725333>

United States, North Carolina, Nash, Swift Creek, on U.S. Rt. 301, 2 mi.n. of Battleboro, William B. Fox, R. K. Godfrey, 1949-07-22, NCSC00014530
<https://portal.torcherbaria.org/portal/collections/individual/index.php?occid=8725335>

United States, Indiana, Cass, About 2 mi. west of New Waverly., Charles C. Deam, 1927-07-28, IND-0069668
<https://portal.torcherbaria.org/portal/collections/individual/index.php?occid=12537662>

United States, Indiana, Allen, about 4 mi northeast of Fort Wayne, Charles C. Deam, 1925-07-02, IND-0069666
<https://portal.torcherbaria.org/portal/collections/individual/index.php?occid=12537664>

United States, Indiana, Jasper, The Kankakee River, about 5 mi. northeast of DeMotte, Charles C. Deam, 1931-07-17, IND-0069679
<https://portal.torcherbaria.org/portal/collections/individual/index.php?occid=12537645>

United States, Virginia, James City, near edge of man-made marsh next to private yard. Marsh on Brascome Blvd., 1460 off of Neck-O-Land Rd., SW of Williamsburg., Joy, Phil; Bruner, L, 1992-10-11, 58715
<https://portal.torcherbaria.org/portal/collections/individual/index.php?occid=7792720>

United States, Virginia, Southampton, Nottoway River Swamp, Mikula, Bernard, 1950-10-28, 37575
<https://portal.torcherbaria.org/portal/collections/individual/index.php?occid=7792873>

United States, South Carolina, Charleston, At upper end of Goose Creek Reservoir. 6 miles east southeast of Summerville., Wilbur H. Duncan, 1943-10-09, NCSC00014547
<https://portal.torcherbaria.org/portal/collections/individual/index.php?occid=8725352>

United States, Illinois, Rock Island, 30 feet north of house located at 1815 16th Street in Rock Island, Marla Thomas, 1996-10-15, AUGIE-02643
<https://midatlanticherbaria.org/portal/collections/individual/index.php?occid=31360677>

United States of America, Indiana, Jasper, Roesler Property, approximately 6 miles south of Wheatfield., Namestnik, S | Roesler, J | Utroske, F, 2009-05-30, 0054761MOR
<https://midatlanticherbaria.org/portal/collections/individual/index.php?occid=4283237>

United States, Indiana, Posey, Twin Swamps Nature Preserve and Gray Estate; located along the N side of County Rd 1400 S and 560 meters E of the intersection with Bonebank Rd, Richard M. Hull, 2022-06-09, IND-0160931
<https://midatlanticherbaria.org/portal/collections/individual/index.php?occid=29898075>
