# Supplementary material for: Beyond air temperature: An examination of leveraging operative temperature space for the discovery of bioinspired thermal design features
Source: PLoS One. 2026 Sep 16;21(9):e0358463. doi: 10.1371/journal.pone.0358463 (PMC13580958; doi:10.1371/journal.pone.0358463)
Supplement: Supplementary file 2 [file pone.0358463.s003.docx]

**Fig 1. k-Distrance plot for air and operative temperature space.**

**Fig 2. Minimum neighbor point sensitivity study. Results shown are for an ε=2.5°C and show stabilization of cluster size starting at a minimum neighbor count of 25.**

**Fig 3. Cluster stability around neighborhood separation parameter for air temperature space. Left hand plots show group geography bounds, right hand plots show clusters in temperature space.**

**Fig 4. Cluster stability around neighborhood separation parameter for operative temperature space.**

**Fig 5.** **Maximum composite 2m air temperature basal distribution of P. heterophylla and air temperature margin outlines based on operative temperature subset population.** Temperature-space distribution groupings outlined in red (A_a_) and orange (B_a_) (top chart). Leaf margin specimen locations are highlighted in triangles in species distribution map by cluster color; outliers are plotting in grey triangles aligned with those ignored during operative temperature assessments. Leaf specimen outline overlays for the temperature-space groupings are plotted and scaled by leaf length (bottom charts denoted “A_a_” and “B_a_”).

**Fig 6. Margin assessment results for ambient temperature groups A_a_ and B_a_ based on operative temperature specimen subset.**
Leaf width (lc) shown on left, average aspect ratio in the middle, and non-dimensionalized average edge radii is shown on right. Error bars reflect 95% confidence interval around value means.

**Table 1. Leaf margin parameters statistics and A-D Normality Test values for air temperature groupings for operative temperature space specimens.**

| **Parameter** | **Grouping** | **N** | **Mean** | **Std. Deviation** | **A-D Statistic** | **A-D *p*-Value** |
| --- | --- | --- | --- | --- | --- | --- |
| Edge Radii | Aa | 60 | 6.68 | 1.45 | 0.52 | 0.17 |
|  | Ba | 26 | 4.99 | 1.42 | 0.26 | 0.70 |
| Aspect Ratio | Aa | 60 | 0.83 | 0.11 | 0.56 | 0.14 |
|  | Ba | 26 | 0.85 | 0.11 | 0.72 | 0.05 |
| Leaf Width (cm) | Aa | 60 | 9.16 | 2.32 | 0.61 | 0.10 |
|  | Ba | 26 | 9.61 | 2.75 | 0.32 | 0.53 |

**Table 2 Leaf margin group comparisons between A_a_ and B_a_ for operative temperature space specimens.**

| **Parameter** | **T-Test df** | **T-stat** | **T-Test p-Value** | **Levine’s df** | **Levine’s**  **f-stat** | **Levine’s**  ***p*-Value** |
| --- | --- | --- | --- | --- | --- | --- |
| Edge Radii | 84 | 4.97 | < 0.01 | 1, 84 | 0.02 | 0.88 |
| Aspect Ratio | 84 | -1.04 | 0.30 | 1, 84 | 0.06 | 0.81 |
| Leaf Width (cm) | 84 | -1.04 | 0.44 | 1. 84 | 2.16 | 0.14 |
