# Supplementary material for: Beyond air temperature: An examination of leveraging operative temperature space for the discovery of bioinspired thermal design features
Source: PLoS One. 2026 Sep 16;21(9):e0358463. doi: 10.1371/journal.pone.0358463 (PMC13580958; doi:10.1371/journal.pone.0358463)
Supplement: Supplementary file 3 [file pone.0358463.s005.docx]

**Subject:** Response to Reviewers – [PONE-D-25-37512]

**Date:** June 18^th^, 2026

Dear Shailender and Reviewers,

Thank you for the in-depth and thorough review of our manuscript *Beyond Air Temperature: An examination of leveraging operative temperature space for the discovery of bioinspired thermal design features*. We have carefully reviewed the comments provided and have worked to address the points raised within the paper. Their insights have been invaluable in reassessing the methods of the study and have providing meaningful feedback that has strengthening the research presented.

In response to the revision decision, we have reworked the study to address previous shortcomings by incorporating improved statistical rigor and expanding the number of specimens incorporated in this work. A significant revision was the calculation of the edge radii parameter to focus on radii with respect to edge deviation rather than the leaf width. This pivot better reflects currently fielded edge structures shown in the newly incorporated Figure 4 and as cited in reference (40). These ‘undulations’ are largely used to propagate turbulent structures near the edges of surfaces as illustrated in (40) and additional references (41)(42)(43). This pivot better reflects the incorporation of this feature in enhancing edge transport in cooling designs.

Another consideration brought up by the reviewers is the applicability of the operative temperature as the output of a modeled leaf rather than a derived climate parameter. The operative temperature parameter developed is intended to serve as an abiotic factor to differentiate thermal environments without treating heat related forcings as completely independent drivers. It is intended to drive investigation along thermal clines rather than explicitly model physiological outcomes. A new subsection, *Limits of the parameter*, has been incorporated in the Discussion section to explicitly discuss the factor and its intended use for a study framework.

Additionally, an extra version of the manuscript has been uploaded this time to address the readability issues multiple reviewers cited by figure placement at the end of the document. This is intended to be the ‘as desired’ placement and size of figures within the text.

We have drafted point-by-point responses to the reviewers’ detailed remarks below. Thank you for consideration and feedback on our submission.

**Sincerely,**

Thomas Slavens
Corresponding Author
University of Oklahoma, College of Engineering
Email: [tslavens@ou.edu](mailto:tslavens@ou.edu)

**Responses to Reviewers – [PONE-D-25-37512]**

| **Reviewer Comment** | **Response** | **Location in revised manuscript** |
| --- | --- | --- |
| **Reviewer 1:** Integrate Broader Literature Comparisons "The manuscript references key studies on leaf thermoregulation, but a more comprehensive comparison with similar herbarium-based analyses (e.g., those using digitized collections for trait evolution) would strengthen the novelty claim." | A new section in the document was introduced covering the use of digital herbarium for this study. A number of references were introduced that used this relatively new digital resource to study species ranges and phenology. | Methods: “Study specimens from digital herbarium” |
| **Reviewer 1:** Refine the Statistical Analysis Approach "The current statistical analysis relies on independent Student’s T-tests to compare morphological parameters between multiple groups (e.g., comparing groups Ao, Bo, Co, and Do). Using multiple T-tests increases the risk of Type I errors (false positives). It is highly recommended to use an Analysis of Variance (ANOVA) followed by an appropriate post-hoc test (such as Tukey's HSD) for multiple comparisons. Additionally, please state whether the data met the assumptions of normality and homogeneity of variances (e.g., via Shapiro-Wilk and Levene's tests); if not, a non-parametric alternative like the Kruskal-Wallis test should be utilized given the varying and sometimes small sample sizes (e.g., n=9 for group Co)." | The statistical assessments for the operative temperature groups have been refined and leverage an appropriate ANOVA and post hoc test to determine significant group differences. | Results: “Results of margin assessments on segregated leaf populations. |
| **Reviewer 1:** Enhance Figure Clarity and Accessibility "Figures such as 7 and 8 effectively illustrate trends in morphological parameters, but they could be improved for better readability. Consider adding color-coding or annotations to clearly distinguish between air temperature and operative temperature groupings (e.g., using distinct symbols or legends)." | Figures have been revamped with higher resolution images along with tables with descriptive statistics of edge radii between groups. Further documentation of graphical information for leaf width and aspect ratio in tabular form to be included in supplemental data set. | Results: “Results of margin assessments on segregated leaf populations.” See updated Figures 11 & 12 and Tables 1 & 2. |
| **Reviewer 1:** Enhance the Presentation of Statistical Results "While Figures 7 and 8 provide a good visual representation of the morphological trends, relying solely on bar charts with error bars makes it difficult for readers to extract precise values. Please consider adding a summary table that explicitly lists the sample size (n), mean, standard deviation, and precise p-values for aspect ratio, leaf width, and non-dimensionalized edge radii across all temperature groupings (both air and operative). This will greatly improve the clarity and rigor of the data presentation." | Descriptive statistics and normality test results for edge radii have been included in Tables 1 & 2. Between group comparison statics information is included in updates within the results section. | Results: “Results of margin assessments on segregated leaf populations.” See updated Figures 11 & 12 and Tables 1 & 2. |
| **Reviewer 2:** The authors should make the connection between leaf-edge morphology and heat-transfer problems more explicit. At present, this connection is understandable, but it is not developed strongly enough. A short explanation is needed to show why the edge of a leaf is important from the viewpoint of boundary-layer behavior, local diffusion, and convective exchange. This would make the transition from botanical observation to bioinspired thermal design more convincing. | Additional information on how the edge radii is related to prior industry work and studies have been added along with an additional figure highlighting the transition in edge serration with a change in radii parameter. Further, adjustment to the non-dimensionalization to be based on the average edge deviation, rather than leaf width, better associates this parameter with edge effect development. Further information was added that highlights studies on the aerodynamic stability and heat transfer impacts leading edge undulations provide (similar in form to the edge margin nodules on the leaf). While these do not directly tie in the radii to heat transfer rates, these do indicate that they have an impact to boundary layer dynamics on the adjacent surfaces. | Methods: “Leaf margin extraction and analysis” |
| **Reviewer 2:** The paper would benefit from a clearer separation of two different results. One result is methodological: operative temperature provides a more detailed and physically meaningful classification of the species range. Another result is morphological: the identified groups show differences in leaf-margin parameters, especially in the characteristic edge radius. These two results are connected, but they should be presented as two consecutive levels of evidence. This would make the logic of the paper clearer. | Additional verbiage within the introduction section has been added to better parse the two results the study directly developed. | Introduction, last paragraph. |
| **Reviewer 2:** The selected DBSCAN search radius of 2.5 °C needs additional explanation. From a heat-transfer point of view, this value is not a direct physical temperature difference between two locations. The clustering is performed in a multidimensional temperature space that includes monthly maximum, minimum, and average temperatures. Therefore, the meaning of a 2.5 °C distance in this space is not self-evident. The authors should explain how this value was selected, whether the temperature variables were normalized or scaled before clustering, and whether the obtained groups remain stable if this parameter is moderately changed. Even a short sensitivity check would make the clustering results more reliable. | The 2.5°C parameter was developed through a sensitivity assessment of both air and operative temperature spaces on the raw values from species basal locations. Results section has been updated to highlight this sensitivity study and identify how the 2.5°C parameter was established. | Results: “Results of cluster-based analysis on leaf specimen segregation” |
| **Reviewer 2:** The limited number of leaf specimens should be discussed more clearly. This limitation is acceptable for a study that tests an idea, but it should be treated explicitly. Some operative-temperature groups contain relatively few specimens, and the material comes from herbarium images collected in different years and probably under different local growth conditions. The authors should comment on how specimen source, collection year, leaf selection, possible damage, leaf age, and local microenvironment may affect the interpretation of the statistical results. | To help strengthen results, further specimens were included in the study where possible. Sans grouping Co, most groups have >20 specimens with their respective clusters. Co is limited by availability in digital herbarium databases and as such may present an incomplete view of species behavior in this location. | Methods: “Study specimens from digital herbarium” |
| **Reviewer 2:** The manuscript does not yet demonstrate a ready engineering solution. It identifies a candidate biological morphology that may be useful for further thermal or diffusive design studies. I do not consider this a serious weakness, because the paper is mainly methodological and exploratory. However, this level of evidence should be formulated clearly, so that the engineering meaning of the results can be stated carefully. | The intent of the method is to allow engineers that are not explicitly engaged in biological or ecological studies an improved abiotic parameter to gauge environmental heat load a species may encounter. Once species are found to inhabit distinctly loaded locations, the intent would be to leverage taxa comparisons between regions to identify physiological or morphological adaptions of interest to model and evaluate further. A pre-exploratory notional configuration of interest based on this leaf edge undulations is cited in (40) and shown in new Figure 4. | Methods: “Leaf edge margin extraction and analysis” |
| **Reviewer 3:** First of all, this paper is a little bit confusing, since the authors do not clearly explain how the operative temperature is calculated. | Details of the operative temperature calculations are captured in the authors prior work cited in reference (5). |  |
| **Reviewer 3:** Moreover, in the view of heat transfer, there should be some kind of assumptions when calculating such operative temperature. How could those assumptions affect the clustering analysis results? | A major assumption of the operative temperature calculation is the magnitude of the surface width for convective heat transfer calculations. New Figure 1 shows a typical diurnal calculation of operative temperature for both a 3 cm and 5 cm surface. This adjustment shows an approximately 3°C increase in peak operative temperature. The 3cm characteristic length was ultimately chosen due to (1) better approximation of transport mechanisms along leaf edges and (2) to ensure that the flow modeled would be within the laminar Reynolds number of the calculation under large swings in ambient wind speeds. | Methods: “Species subgroup development based on temperature space” |
| **Reviewer 3:** Lastly, the current version is more like a botanical paper in my view, and how would these finds by operative temperature be utilized for bioinspired thermal design ? Please give a practical example at least. | The intent of this work is to help bridge gaps between biological disciplines and engineering design. Much of the focus of the study is to provide a framework to allow a thermal designer to investigate where a species of interest may be identified by providing a singular parameter that encompasses more of the heat loadings a species may encounter. In developing this framework, the bulk of the research has been centered on developing and interrogating the operative temperature parameter in providing a context from which further morphological studies may be launched. A pre-exploratory notional configuration of interest based on this leaf edge undulations is cited in (40) and shown in new Figure 4. | Methods: “Leaf edge margin extraction and analysis” |
| **Reviewer 4:** The manuscript repeatedly suggests that operative temperature enables identification of thermally relevant morphological features for engineering design. However, the study demonstrates only weak correlations between cluster assignments and simple geometric descriptors. There is no demonstration that the identified features (e.g., reduced edge radii) meaningfully affect heat transfer performance. The conclusion that observed trends relate to early-season thermal adaptation rather than peak heat load is speculative. In its current form, the manuscript does not establish a causal or mechanistic link between operative temperature and functional morphology. | The intent of the study is to demonstrate the operative temperature parameter as a metric of merit in helping to frame the aggressiveness of heat load a taxa may encounter in the field. The use of the margin assessment of a distributed species is intended to gauge the effectiveness of this parameter in comparison to using air temperature alone. Additional explanation of prior studies and industrial development of convective apparatus leveraging leading edge radii augmentation has been provided included in the methods section.  With the updated analysis and results, the authors have adjusted the observations and conclusions to support further studies down cold-weather dynamics of impacts to leaf margin serration as supported by Figures 9, 14, and 15. From Figure 9, the scatter plot illustrates Maximum To overlap between groups B_o_ and A_o_, and between groups B_o_ and C_o_. In Figures 14 and 15, it is observed that differences in temperatures between the clusters is only observed in early months between B_o_ and A_o_. These results would not support that the trend of reducing margin edge radii would be strongly driven by mid-season peak temperatures. | Discussion: “Margin assessments of *P. heterophylla* samples”  Conclusions. |
| **Reviewer 4:** The analysis is based on 73 manually selected leaves, drawn from heterogeneous herbarium sources spanning long time periods and unknown environmental conditions. The dataset is small relative to the variability inherent in biological morphology. No control exists for confounding variables (age, hydration state, light exposure, damage). The authors explicitly acknowledge these inconsistencies but proceed without addressing their impact quantitatively. This raises serious concerns that the reported trends may reflect sampling artifacts rather than biological signal. | Additional specimens (103) and updated descriptions of the controls of the specimens to account for leaf maturity and collection are provided in a new methods section specifically on the utilization of herbarium species for this study. | Methods: “Study specimens from digital herbarium” |
| **Reviewer 4:** Leaf outlines are manually digitized and resampled however; no validation of repeatability or inter-operator consistency is provided. The selection of “clean” leaves introduces subjective bias. The downstream metric (edge radius) is highly sensitive to boundary definition. Without uncertainty quantification, the reliability of the extracted geometric parameters is questionable. | A repeatability study was conducted early-on within the research to assess digitization variability. The assessment was completed across two samples with five replicate extractions. | Methods: “Leaf edge margin extraction and analysis” |
| **Reviewer 4:** The statistical treatment is minimal and insufficient for the claims made. Assessment relies on simple t-tests without: effect sizes, multiple comparison correction, assessment of distribution assumptions. There is no multivariate analysis despite multiple correlated morphological variables nor demonstration that observed differences are practically meaningful, even when statistically significant. Overall, the statistical analysis does not meet the standard expected for a data-driven study. | The statistical assessments for the operative temperature groups have been refined and leverage an appropriate ANOVA and post hoc test to determine significant group differences. While multiple parameters are examined, the parameters do not represent multiple controlled variables in which to explicitly leverage multivariate statistics to address population differences. The premise of the development of the operative temperature is to eliminate the multiple climate variables that would need to be taken into consideration when examining taxa variation along thermal clines.  Morphological variables examined were not found to be correlative within the groups studied (R2 = 0.002 – 0.03, r = 0.08-0.2). Linear plots are contained in the supplemental data package. | Results: “Results of margin assessments on segregated leaf populations.”  Supplemental data package, tab “Margin Correlations” |
| **Reviewer 4:** The DBSCAN clustering parameters (ε = 2.5°C, minPts = 15) appear arbitrarily selected. However, no sensitivity analysis is provided. There is no validation that the clusters are stable or physically meaningful. Not the least, no comparison with alternative clustering methods  Given that the central claim depends on identifying “more granular clusters,” the absence of robustness analysis is a major flaw. | The 2.5°C parameter was developed through a sensitivity assessment of both air and operative temperature spaces on the raw values from species basal locations. Results section has been updated to highlight this sensitivity study and identify how the 2.5°C parameter was established. | Results: “Results of cluster-based analysis on leaf specimen segregation” |
| **Reviewer 4:** The operative temperature formulation assumes a massless, fixed-size leaf with no physiological regulation. Real leaves actively regulate temperature via transpiration and stomatal control. Ignoring these effects undermines the physical realism of the metric. As a result, it is unclear whether the operative temperature used here meaningfully represents the thermal environment experienced by the organism. | The operative temperature is intended to be interpreted as an abiotic parameter rather than a true prediction of leaf thermal performance. As such, an increase of assumptions to the operative temperature parameter that includes physiological operations of the leaves may introduce biases in the calculation that masks the austerity of the local environment. Further, no clear baseline transpiration rate or configuration is referenced in studies that would bias the parameter would be practical to incorporate. |  |
| **Reviewer 4:** The manuscript is framed around bioinspired thermal design, yet no engineering model, simulation, or experiment is presented. There is no performance metric (e.g., heat transfer coefficient, diffusion enhancement) is evaluated. The discussion of applications remains speculative. This significantly weakens the contribution, as the work does not extend beyond ecological correlation into actionable engineering insight. | The intent of the study is to gauge the merit of the operative temperature metric to identify species morphological variation along a heat load gradient. The method is intended to allow a cooling designer a short-hand means to identify where a species may exhibit phenotypic plasticity of interest that can be further studied, abstracted, and incorporated into a thermal design later based on similar load/conditions. Further work is intended to leverage this study to find these morphologies of interest and integrate them into thermal designs.  A pre-exploratory notional configuration of interest based on this leaf edge undulations is cited in (40) and shown in new Figure 4. | Methods: “Leaf edge margin extraction and analysis” |
| **Reviewer 4:** Minor Concerns: The manuscript incorrectly uses “inter-species” when analyzing a single species. This should be corrected. Several sentences are awkward or unclear (e.g., “poses to offer”), and the manuscript would benefit from careful editing. Many figures (particularly overlay plots) are qualitative and difficult to interpret quantitatively. The use of a Google Docs link for data sharing is not appropriate for long-term reproducibility. Last by not the least, some references are repeated unnecessarily. | The authors have worked back through the manuscript to better align the clarity and intent of the text. Work to revamp figures and provide tabular data for key parameters has been completed to better describe results of the study. | Abstract  Figures 11 & 12  Tables 1 & 2 |
| **Reviewer 5:** 73 digital leaf specimens are investigated. It would be better to give a brief introduction of these specimens. How you chose these specimens. | An updated section detailing herbarium specimens and selection has been added to the text. | Methods: “Study specimens from digital herbarium” |
| **Reviewer 5:** how many specimens selected in different regions. | A total of 103 specimens were selected across the entirety of the basal region of the study species. While specific numbers per cluster region would be optimal, availability of specimens within the digital databases are highly variable. As such the study targeted to have at least 20 specimens per cluster, but did not meet that target for grouping Co which only had 11. Specimen counts within clusters are provided in Tables 1 & 2 and Figures 8 & 10. | Tables 1 & 2  Figures 8 & 10 |
| **Reviewer 5:** the authors mentioned the thermal design, however, the results only show the relation between temperature and characteristic radii. Besides temperature, the thermal design may also relate to thermal environment like the wind, rain etc. | Further studies to incorporate local hydrodynamics have been identified for future work. Once appropriate parameters are developed that do not rely on *a priori* assumptions of physiological performance, these could be further tied into the notional design space. |  |
| **Reviewer 5:** the statistical method, like T-test, used in this study should be described in detail. | More details into the statistical tests between groups has been provided in-text in the results section. Descriptive statistics have been added to Tables 1 & 2. | Results: “Results of margin assessments on segregated leaf populations.”  Tables 1 & 2 |
| **Reviewer 6:** The manuscript introduces operative temperature as a more comprehensive metric, but its physical meaning and limitations are not clearly discussed. The assumption of a “massless surface” used for its calculation is particularly problematic and requires justification. It remains unclear how well this metric represents real leaf thermodynamics, especially under transient and heterogeneous environmental conditions. | As the intent of the operative temperature is poised to describe heat load aspect of the environment a taxa resides in, inclusion of specific physiology would a priori assume some significant feature that may be an adaptively significant phenotype. While the parameter assumes a base parameter for convective and radiative balance with the environment, physiological features such as leaf mass or stomata response rates would shape the operative temperature to implicitly reflect the environment from the perspective of species with these types of features. The parameter is intended to be used as an environmental reference that would indicate where further taxa-specific research would be valuable. |  |
| **Reviewer 6:** The study relies entirely on calculated operative temperatures without validation. How sensitive the operative temperature is to input parameters such as wind speed, radiation intensity, or surface properties?  Without such validation, it is difficult to assess whether the observed differences between clusters are physically meaningful. | Figure 1 was added to the paper to illustrate how the parameter varies during a diurnal period along with a comparison to a field observation. The field observation was based on an IR measurement of a construction paper specimen to closely approximate the analytical model the operative temperature is based on. While not an in-depth study, the point observation gives credence to the magnitude of the parameter to have merit in describing the local heat load a taxa may experience. | Methods: “Species subgroup development based on temperature space”  Figure 1 |
| **Reviewer 6:** The main argument is that operative temperature provides "greater granularity". However, increased clustering resolution alone does not demonstrate improved physical relevance. The manuscript does not convincingly show that operative temperature leads to better or more physically grounded interpretation of heat transfer phenomena. | Prior studies of leaf morphology variation has typically used latitude or average air temperatures to investigate shifts in phenotypes (16) (11) (12) (14) (15). These factors do not directly account for the impacts of solar irradiance and convective transfer. The developed massless operative temperature is a step in the direction to incorporate these transfer mechanisms within a singular parameter that can be used to differentiate regions of a species basal range without the necessity of treating these inter-dependent parameters as a separate vector space. The present study highlights the use of this parameter to highlight 2 intermediary regions with behavior that highlights a level of morphological transition along the margins of the current study species. |  |
| **Reviewer 6:** The discussion of results remains largely qualitative and does not explicitly link observed trends to heat transfer mechanisms. While references are made to convection and diffusion processes, no quantitative analysis is provided.  From a thermal engineering perspective, the study does not establish how the identified differences influence heat transfer performance. | The intent of the study is to primarily gauge the merit of leveraging the operative temperature parameter to highlight where taxa’s of interest may reside. The goal of the work is to substantiate the use of this parameter to enable thermal engineers who are not intimate with ecological studies to query and identify where to start a morphological study. |  |
| **Reviewer 6:** The observed variations in edge radius and aspect ratio are relatively small. The manuscript does not provide a clear analysis of whether these differences are significant from a heat transfer standpoint. Specifically, it would strengthen the paper considerably to address whether the observed differences in edge radius, e.g. in the order of 10–15% between groups, correspond to any meaningful change in convective heat transfer coefficient or Nusselt number. Without such an assessment, the connection between the biological findings and thermal engineering applications remains speculative. | Updates to the edge radii non-dimensionalization to focus on the radii relative to local edge deviation highlighted a larger magnitude in variation between clusters.   The burden of establishing thermal relevance of these differences in edge radii is intended for follow-on work focusing on evaluating transport dynamics of the structures and derivation to thermal design through functional analogy.  The current study is intended to provide an enhanced ecological framework to highlight these structures while leaving mechanism and abstraction as follow-on engineering activities. | Methods: “Leaf edge margin extraction and analysis”, updated Figure 4. |
