# Supplementary material for: Beyond air temperature: An examination of leveraging operative temperature space for the discovery of bioinspired thermal design features
Source: PLoS One. 2026 Sep 16;21(9):e0358463. doi: 10.1371/journal.pone.0358463 (PMC13580958; doi:10.1371/journal.pone.0358463)
Supplement: Supplementary file 4 [file pone.0358463.s006.docx]

**Subject:** Response to Reviewers – [PONE-D-25-37512]

**Date:** August 23^rd^, 2026

Dear Mohammad and Reviewers,

Thank you for the in-depth and thorough review of our revised manuscript *Beyond Air Temperature: An examination of leveraging operative temperature space for the discovery of bioinspired thermal design features*, with a special thank you to Reviewer 2 for the additional feedback through the manuscript. We have carefully reviewed the comments provided and have worked to address the points raised with the manuscript. The feedback is greatly appreciated.

We have reworked the study to address the concerns with the clustering method sensitivity to temperature space scale differences between the air temperature and operative temperature space groups. The revision treats the two spaces independently and utilizes a 90% capture criteria to identify the neighborhood search radius (ε) for each regime. Results of the evolution of captured and outlier data is shown a new Figure 5 with supporting plots of k-Distance and minimum neighbor sensitivity added into new Supplemental Document Figures 1 and 2 respectively. Further, cluster evolution plots have been adjusted to better highlight species location points that are included with specific clusters and which are still considered outlier data (Figure 6 and 7).

Additional work was performed to ensure accounting of leaf specimens through the evolution of the study (Figure 12). Here specimens are culled due to smaller sizes indicating non-mature specimens and further culled as they are not included within cluster spatial zones. An secondary study that included just the specimens used for the operative temperature space assessments were used under air temperature segregation (see Supplement Figures 5 and 6, Tables 1 and 2) to ensure the results of the main study were not significantly impacted due to the loss of specimens during cluster exclusions. The results of this study remained consistent to the full N_a_=98 assessment. Inconsistencies in the statistics were also corrected. For the one population that failed normality criteria (leaf widths of group A_a_), it is noted in the text and a non-parametric test is used to compare population mean to B_a_.

Additionally, an extra version of the manuscript with revisions has been uploaded along with a revision with images to ensure that errors associated with the changing document has been addressed.

We have drafted point-by-point responses to the reviewers’ detailed remarks below. Thank you for consideration and feedback on our submission.

**Sincerely,**

Thomas Slavens
Corresponding Author
University of Oklahoma, College of Engineering
Email: [tslavens@ou.edu](mailto:tslavens@ou.edu)

**Responses to Reviewers – [PONE-D-25-37512]**

| **Reviewer Comment** | **Response** | **Location in revised manuscript** |
| --- | --- | --- |
| **Reviewer 1:** Small advice to author: The authors did not follow your statistical recommendation perfectly for the two-group comparison (Aa vs Ba). They retained an independent t-test there, but statistically that is completely acceptable because only two groups are being compared. Your concern was primarily about performing many pairwise t-tests across four groups, and they corrected exactly that by switching to ANOVA with Tukey HSD for the operative-temperature analysis. So I would not press this point further.  The authors have responded satisfactorily to my comments. The manuscript has been substantially improved, particularly with respect to the statistical analysis, inclusion of descriptive statistics, expansion of the literature review, and improved figure presentation. I appreciate the authors' efforts in revising the manuscript and have no further major concerns. I RECOMMEND ACCEPTANCE. | Thank you for the time to review our paper and the feedback. |  |
| **Reviewer 2:** Robustness of the DBSCAN result  This is the main remaining problem.  The clustering is performed in a 24-dimensional space using raw, unscaled temperature values. Operative temperature has a wider numerical range than 2 m air temperature, but the same DBSCAN radius, ε = 2.5°C, is used for both spaces.  The authors obtain 2 clusters in air-temperature space and 4 clusters in operative-temperature space. However, a more dispersed point cloud can naturally produce more clusters with the same fixed ε. DBSCAN, unfortunately, does not know that one space is more physically meaningful than another.  Therefore, the authors should show that the result is not mainly a scaling effect. At minimum, they should:  • repeat clustering after standardization of the 24 variables, or quantitatively justify why the same absolute ε is appropriate;  • report the number and percentage of noise points;  • show stability under moderate changes around ε = 2.5°C;  • clarify whether minPts = 30 was fixed and whether a modest change of this value affects the result.  The model sensitivity also deserves a short comment. Changing the characteristic length from 3 cm to 5 cm changes peak operative temperature by about 3°C, which is already larger than ε = 2.5°C. It is not necessary to repeat the whole study, but the possible effect on cluster boundaries should be discussed.  Without this check, the central 2-versus-4 cluster result remains not fully safe. | The authors thank Reviewer 2 for the further insight in utilization of the DBSCAN method in identifying clusters in temperature spaces.  In reflection of utilization of the DBSCAN method, the authors have pivoted to adjust scan radii to be individually tailored to each temperature dimensional space rather than a single target value based. As pointed out by the reviewer, the DBSCAN is sensitive to the scale of the space it is executed within. Since this study is focused on the ability of the inclusion of a heat flux balance to broaden the temperature space of a species to identify inter-species variations of interest, it did make more sense to alter the DBSCAN search radius to each temperature space rather than non-dimensionalize spaces to parity. The new search radii were based on 90% capture of the species points and supported by the factors position in a k-distance graph. Sensitivity to neighbor count is also provided with the selected neighbor count being chosen as 35 for both temperature spaces. Additionally, a sensitivity to the chosen ε values were conducted for both air and operative temperature spaces showing a moderate robustness of ~0.1°C for air and 0.15°C for operative space.  A final paragraph was added to address the magnitude impacts of changes in leaf width assumptions relative to shifts in temperature space. As observed in Fig. 1, increase in l_c_ pushes the maximum temperature calculated due to the increase in received solar radiation to the surface and the reduced convective transfer over a larger flat plate. This shift in peak could ultimately provide larger differences across population areas increasing group resolution from the cluster analysis. A reduction in l_c_ would work opposite to this keeping partitions closer to as observed in air temperature space. A further study of a species with relatively thin leaves (willow or pine) and wide leaves (maple) would be of interest to provide insight into this relationship. | Figure 5 Supplement 3, Figures 1, 2, 3, and 4  Discussion section, “Limitations of the parameter”, last paragraph |
| **Reviewer 2:** Accounting for specimens and checking statistics  The manuscript states that 103 specimens were analyzed.  For the air-temperature groups, the reported sample sizes are 67 + 25 = 92. Thus, 11 specimens are absent from this analysis.  For the operative-temperature groups, the sample sizes are 24 + 24 + 11 + 25 = 84. Thus, 19 specimens are absent.  Probably these specimens belong to DBSCAN noise, but the leaves should not disappear silently. The manuscript should state clearly:  • how many specimens were assigned to every cluster;  • how many were excluded as noise;  • whether there were other exclusions;  • whether the air-temperature and operative-temperature analyses use different subsets.  Because the two analyses use 92 and 84 specimens, respectively, part of their difference may come from sample composition. A comparison using the common subset assigned in both analyses would be useful.  The statistical tables also need careful checking.  The aspect-ratio means for the operative-temperature groups are reported as 7.21, 6.21, 6.47, and 4.95. These values appear to duplicate the edge-radius values and are inconsistent with the figure, where aspect ratio is around 0.8.  The manuscript also says that all groups satisfied normality, but at least two Anderson-Darling results have p = 0.04. With alpha = 0.05, this is not normality confirmation.  The authors should verify all sample sizes, means, standard deviations, test statistics, degrees of freedom, and p-values. Exact p-values, effect sizes, and confidence intervals for the main comparisons would be preferable. | The full 103 represented the total number of digitized specimens. Clarity has been added to the text to show how this population was reduced first by leaf width standards to ensure mature and representative leaves. Subsequent reductions were then made based on the occurrence of the specimen within an identified temperature-driven spatial cluster region. This evolution is highlighted in new Figure 12 and addressed in the results and discussion sections.  A secondary assessment was performed to address inconsistencies of results of the Na = 98 of the air temperature groupings versus the No = 86 of the operative temperature groupings. This study repeated the assessment of the air temperature group, but excluded those specimens that were not used in the operative temperature assessment. Results are included in supplement 3 and are consistent with the full Na = 98 analysis.  Tables have been updated to represent current population assessments and checked for consistency with figures. With the exception of air groupings leaf width, all Anderson-Darling groups are >= 0.5. For the exception (air groupings leaf width comparison), a non-parametric test is used to compare these groups (Wilcoxon rank sum test). | Figure 12, Supplementary Figure 5 and supplementary Table 1. |
| **Reviewer 2:** The conclusions are still a little more enthusiastic than the data  The authors are perhaps a little too enthusiastic here. The data show an interesting association, but they do not yet demonstrate the proposed mechanism. It also shows that operative-temperature space can produce 4 environmental groups instead of 2 under the selected DBSCAN settings.  It does not directly prove that:  • operative temperature caused the morphology;  • the morphology is specifically a heat-transfer adaptation;  • cool-season conditions are the causal driver;  • summer heat load is not important;  • the measured radius produces an engineering heat-transfer benefit.  The strongest morphological difference is mainly between the most separated groups, Ao and Do. The intermediate groups Bo and Co are not consistently distinguished by the main edge-radius parameter. In addition, the air-temperature grouping already gives a clear morphological difference.  Therefore, the manuscript demonstrates greater environmental granularity more convincingly than greater morphological discrimination.  Terms such as “heat-transfer-based morphological adaptations” should be replaced by more careful wording, for example “candidate morphological features associated with different regional thermal environments.” The cool-season interpretation should be presented as a hypothesis consistent with the results, not as an established mechanism. | Conclusions, both within the abstract and the conclusion section have been couched to the explicit results. The conclusions are focused on the intent of the study to demonstrate the effectivity of population segregation in operative temperature space provides a higher descriptive framework than use of air temperature alone.  Further work, as discussed in the “Limitations of the Parameter” section, will be necessary in the future to refine the parameter use and correlate to other studies to help support thermal load driven claims of morphological adaptions. | Revised Abstract and Conclusion sections.  Edit to the Discussion section “Margin assessments of *P. heterophylla* samples”, paragraph 4.  “Limitations of the Parameter”, paragraph 1. |
| **Reviewer 2:** Technical preparation  Before resubmission, the authors should update all fields and cross-references in the Word document and then inspect both the Word and PDF versions.  At present, some figures have duplicated references, one valid and one inherited from the previous version. After field updating, these produce messages such as:  Error: Reference source not found.  All obsolete fields, invalid figure references, duplicated cross-references, and old manuscript identifiers should be removed or corrected.  Word fields should not be allowed to make independent scientific decisions in the final PDF. | Fields have been updated for final version; tracked changes version should be only for author review and the final clean version is also provided to ensure intended status of the revision is documented. | See “PLOSOne_BeyondAmbientAirTemp_Revision-2-Manuscript_w-Figures.docx” |
| **Reviewer 6:** All questions and doubts have been explained and addressed. No further action is required. The paper is now ready to be accepted. | Thank you for the time to review our paper and the feedback. |  |
