# Supplementary material for: Beyond air temperature: An examination of leveraging operative temperature space for the discovery of bioinspired thermal design features
Source: PLoS One. 2026 Sep 16;21(9):e0358463. doi: 10.1371/journal.pone.0358463 (PMC13580958; doi:10.1371/journal.pone.0358463)
Supplement: Peer review history

# Peer review: Decision Letter 0

**DOI:** 10.1371/journal.pone.0358463.r001

**Authors:** Shailender Verma

6 May 2026

Dear Dr. Slavens,

Thank you for submitting your manuscript to PLOS ONE. After careful consideration, we feel that it has merit but does not fully meet PLOS ONE’s publication criteria as it currently stands. Therefore, we invite you to submit a revised version of the manuscript that addresses the points raised during the review process.

Please submit your revised manuscript by Jun 20 2026 11:59PM. If you will need more time than this to complete your revisions, please reply to this message or contact the journal office at plosone@plos.org. When you're ready to submit your revision, log on to https://www.editorialmanager.com/pone/ and select the 'Submissions Needing Revision' folder to locate your manuscript file.

- A letter that responds to each point raised by the academic editor and reviewer(s). You should upload this letter as a separate file labeled 'Response to Reviewers'.
- A marked-up copy of your manuscript that highlights changes made to the original version. You should upload this as a separate file labeled 'Revised Manuscript with Track Changes'.
- An unmarked version of your revised paper without tracked changes. You should upload this as a separate file labeled 'Manuscript'.

If you would like to make changes to your financial disclosure, please include your updated statement in your cover letter. Guidelines for resubmitting your figure files are available below the reviewer comments at the end of this letter.

If applicable, we recommend that you deposit your laboratory protocols in protocols.io to enhance the reproducibility of your results. Protocols.io assigns your protocol its own identifier (DOI) so that it can be cited independently in the future. For instructions see: https://journals.plos.org/plosone/s/submission-guidelines#loc-laboratory-protocols. Additionally, PLOS ONE offers an option for publishing peer-reviewed Lab Protocol articles, which describe protocols hosted on protocols.io. Read more information on sharing protocols at https://plos.org/protocols?utm_medium=editorial-email&utm_source=authorletters&utm_campaign=protocols.

As the corresponding author, your ORCID iD is verified in the submission system and will appear in the published article. PLOS supports the use of ORCID, and we encourage all coauthors to register for an ORCID iD and use it as well. Please encourage your coauthors to verify their ORCID iD within the submission system before final acceptance, as unverified ORCID iDs will not appear in the published article. *Only*  the individual author can complete the verification step; PLOS staff *cannot*  verify ORCID iDs on behalf of authors.

We look forward to receiving your revised manuscript.

Kind regards,

Shailender Kumar Verma, Ph.D.

Academic Editor

PLOS One

Journal requirements:

When submitting your revision, we need you to address these additional requirements.

1. Please ensure that your manuscript meets PLOS ONE's style requirements, including those for file naming. The PLOS ONE style templates can be found at

https://journals.plos.org/plosone/s/file?id=wjVg/PLOSOne_formatting_sample_main_body.pdf and

https://journals.plos.org/plosone/s/file?id=ba62/PLOSOne_formatting_sample_title_authors_affiliations.pdf

2. Please note that PLOS One has specific guidelines on code sharing for submissions in which author-generated code underpins the findings in the manuscript. In these cases, we expect all author-generated code to be made available without restrictions upon publication of the work. Please review our guidelines at https://journals.plos.org/plosone/s/materials-and-software-sharing#loc-sharing-code and ensure that your code is shared in a way that follows best practice and facilitates reproducibility and reuse.

If the reviewer comments include a recommendation to cite specific previously published works, please review and evaluate these publications to determine whether they are relevant and should be cited. There is no requirement to cite these works unless the editor has indicated otherwise.

[Note: HTML markup is below. Please do not edit.]

Reviewers' comments:

Reviewer's Responses to Questions

**Comments to the Author**

1. Is the manuscript technically sound, and do the data support the conclusions?

Reviewer #1: Partly

Reviewer #2: Partly

Reviewer #3: Partly

Reviewer #4: No

Reviewer #5: Partly

Reviewer #6: Yes

**********

2. Has the statistical analysis been performed appropriately and rigorously?

Reviewer #1: No

Reviewer #2: No

Reviewer #3: No

Reviewer #4: No

Reviewer #5: No

Reviewer #6: N/A

**********

3. Have the authors made all data underlying the findings in their manuscript fully available?

The PLOS Data policy

Reviewer #1: Yes

Reviewer #2: No

Reviewer #3: No

Reviewer #4: No

Reviewer #5: No

Reviewer #6: Yes

**********

4. Is the manuscript presented in an intelligible fashion and written in standard English?

Reviewer #1: Yes

Reviewer #2: Yes

Reviewer #3: Yes

Reviewer #4: No

Reviewer #5: Yes

Reviewer #6: Yes

**********

Reviewer #1: 1. Integrate Broader Literature Comparisons "The manuscript references key studies on leaf thermoregulation, but a more comprehensive comparison with similar herbarium-based analyses (e.g., those using digitized collections for trait evolution) would strengthen the novelty claim."

2. Refine the Statistical Analysis Approach "The current statistical analysis relies on independent Student’s T-tests to compare morphological parameters between multiple groups (e.g., comparing groups Ao, Bo, Co, and Do). Using multiple T-tests increases the risk of Type I errors (false positives). It is highly recommended to use an Analysis of Variance (ANOVA) followed by an appropriate post-hoc test (such as Tukey's HSD) for multiple comparisons. Additionally, please state whether the data met the assumptions of normality and homogeneity of variances (e.g., via Shapiro-Wilk and Levene's tests); if not, a non-parametric alternative like the Kruskal-Wallis test should be utilized given the varying and sometimes small sample sizes (e.g., n=9 for group Co)."

3. Enhance Figure Clarity and Accessibility "Figures such as 7 and 8 effectively illustrate trends in morphological parameters, but they could be improved for better readability. Consider adding color-coding or annotations to clearly distinguish between air temperature and operative temperature groupings (e.g., using distinct symbols or legends)."

4. Enhance the Presentation of Statistical Results "While Figures 7 and 8 provide a good visual representation of the morphological trends, relying solely on bar charts with error bars makes it difficult for readers to extract precise values. Please consider adding a summary table that explicitly lists the sample size (n), mean, standard deviation, and precise p-values for aspect ratio, leaf width, and non-dimensionalized edge radii across all temperature groupings (both air and operative). This will greatly improve the clarity and rigor of the data presentation."

Reviewer #2: The manuscript addresses an interesting and non-trivial idea. In my opinion, the strongest part of the paper is the attempt to move the search for bioinspired thermal-design features from a simple climatic description to a heat-transfer-based description of the environment. This is a correct and important shift. A biological surface is not exposed to air temperature alone. It is exposed to a combined thermal load formed by solar radiation, convection, and thermal emission. Therefore, the use of operative temperature is physically more meaningful than the use of ambient air temperature as the only classifying parameter.

This point is, in my view, the main value of the manuscript. The authors do not simply introduce another empirical climatic variable. They try to build a search space that is more consistent with the real heat balance of a biological surface. This is a good idea, and the results generally support it. Clustering based on operative temperature gives a more detailed subdivision of the Populus heterophylla range than clustering based on 2 m air temperature. The subsequent analysis of leaf-margin geometry also gives a plausible indication that the proposed metric can reveal morphological differences that may be relevant to thermal or diffusive exchange.

At the same time, the manuscript should be strengthened in several points.

1. The authors should make the connection between leaf-edge morphology and heat-transfer problems more explicit. At present, this connection is understandable, but it is not developed strongly enough. A short explanation is needed to show why the edge of a leaf is important from the viewpoint of boundary-layer behavior, local diffusion, and convective exchange. This would make the transition from botanical observation to bioinspired thermal design more convincing.

2. The paper would benefit from a clearer separation of two different results. One result is methodological: operative temperature provides a more detailed and physically meaningful classification of the species range. Another result is morphological: the identified groups show differences in leaf-margin parameters, especially in the characteristic edge radius. These two results are connected, but they should be presented as two consecutive levels of evidence. This would make the logic of the paper clearer.

3. The selected DBSCAN search radius of 2.5 °C needs additional explanation. From a heat-transfer point of view, this value is not a direct physical temperature difference between two locations. The clustering is performed in a multidimensional temperature space that includes monthly maximum, minimum, and average temperatures. Therefore, the meaning of a 2.5 °C distance in this space is not self-evident. The authors should explain how this value was selected, whether the temperature variables were normalized or scaled before clustering, and whether the obtained groups remain stable if this parameter is moderately changed. Even a short sensitivity check would make the clustering results more reliable.

4. The limited number of leaf specimens should be discussed more clearly. This limitation is acceptable for a study that tests an idea, but it should be treated explicitly. Some operative-temperature groups contain relatively few specimens, and the material comes from herbarium images collected in different years and probably under different local growth conditions. The authors should comment on how specimen source, collection year, leaf selection, possible damage, leaf age, and local microenvironment may affect the interpretation of the statistical results.

5. The manuscript does not yet demonstrate a ready engineering solution. It identifies a candidate biological morphology that may be useful for further thermal or diffusive design studies. I do not consider this a serious weakness, because the paper is mainly methodological and exploratory. However, this level of evidence should be formulated clearly, so that the engineering meaning of the results can be stated carefully.

General remark:

The manuscript is not very comfortable to read in its present form. The figures are placed separately from the corresponding text, and the reader has to move constantly between the text and the end of the manuscript. This is especially inconvenient here, because the figures are not secondary illustrations. They carry the main logic of the study: maps, clusters, leaf outlines, and statistical comparisons must be read together with the text. I recommend placing the figures close to the relevant paragraphs or adding a compact workflow figure that shows the full sequence of the method: climate data, operative-temperature calculation, clustering, assignment of herbarium specimens, leaf-margin extraction, comparison of morphological parameters, and bioinspired interpretation.

Overall, I think that the manuscript is suitable for publication after revision. The paper presents an interesting idea, and this idea receives reasonable preliminary confirmation. The main contribution is the use of a more physically justified thermal metric for bioinspired screening. The suggested revisions are intended to improve the clarity, robustness, and readability of the manuscript, without changing its exploratory character.

Best regards

Reviewer #3: This study presents a method to use the operative temperature (incorporating convective and radiative heat transfer) rather than ambient air temperature for clustering analysis of Populus heterophylla populations, and analyzes leaf margin morphology (aspect ratio, width, edge radius) across identified subgroups.

First of all, this paper is a little bit confusing, since the authors do not clearly explain how the operative temperature is calculated.

Moreover, in the view of heat transfer, there should be some kind of assumptions when calculating such operative temperature. How could those assumptions affect the clustering analysis results?

Lastly, the current version is more like a botanical paper in my view, and how would these finds by operative temperature be utilized for bioinspired thermal design ? Please give a practical example at least.

Reviewer #4: The manuscript proposes using an operative temperature metric (incorporating radiative and convective effects) to cluster ecological niches and infer thermally relevant morphological adaptations for bioinspired design. The approach is applied to Populus heterophylla, and leaf morphology is analyzed across temperature-defined clusters.

While the premise is potentially interesting, the manuscript in its current form is not sufficiently rigorous to support its claims. The work remains largely correlative, methodologically underdeveloped, and overinterpreted, with limited statistical robustness and no direct validation of the proposed engineering relevance.

Overall Recommendation: Major Revision (borderline reject)

Concerns are presented as followed:

1. The manuscript repeatedly suggests that operative temperature enables identification of thermally relevant morphological features for engineering design. However, the study demonstrates only weak correlations between cluster assignments and simple geometric descriptors. There is no demonstration that the identified features (e.g., reduced edge radii) meaningfully affect heat transfer performance. The conclusion that observed trends relate to early-season thermal adaptation rather than peak heat load is speculative. In its current form, the manuscript does not establish a causal or mechanistic link between operative temperature and functional morphology.

2. The analysis is based on 73 manually selected leaves, drawn from heterogeneous herbarium sources spanning long time periods and unknown environmental conditions. The dataset is small relative to the variability inherent in biological morphology. No control exists for confounding variables (age, hydration state, light exposure, damage). The authors explicitly acknowledge these inconsistencies but proceed without addressing their impact quantitatively. This raises serious concerns that the reported trends may reflect sampling artifacts rather than biological signal.

3. Leaf outlines are manually digitized and resampled however; no validation of repeatability or inter-operator consistency is provided. The selection of “clean” leaves introduces subjective bias. The downstream metric (edge radius) is highly sensitive to boundary definition. Without uncertainty quantification, the reliability of the extracted geometric parameters is questionable.

4. The statistical treatment is minimal and insufficient for the claims made. Assessment relies on simple t-tests without: effect sizes, multiple comparison correction, assessment of distribution assumptions. There is no multivariate analysis despite multiple correlated morphological variables nor demonstration that observed differences are practically meaningful, even when statistically significant. Overall, the statistical analysis does not meet the standard expected for a data-driven study.

5. The DBSCAN clustering parameters (ε = 2.5°C, minPts = 15) appear arbitrarily selected. However, no sensitivity analysis is provided. There is no validation that the clusters are stable or physically meaningful. Not the least, no comparison with alternative clustering methods

Given that the central claim depends on identifying “more granular clusters,” the absence of robustness analysis is a major flaw.

6. The operative temperature formulation assumes a massless, fixed-size leaf with no physiological regulation. Real leaves actively regulate temperature via transpiration and stomatal control. Ignoring these effects undermines the physical realism of the metric. As a result, it is unclear whether the operative temperature used here meaningfully represents the thermal environment experienced by the organism.

7. The manuscript is framed around bioinspired thermal design, yet no engineering model, simulation, or experiment is presented. There is no performance metric (e.g., heat transfer coefficient, diffusion enhancement) is evaluated. The discussion of applications remains speculative. This significantly weakens the contribution, as the work does not extend beyond ecological correlation into actionable engineering insight.

8. Minor Concerns: The manuscript incorrectly uses “inter-species” when analyzing a single species. This should be corrected. Several sentences are awkward or unclear (e.g., “poses to offer”), and the manuscript would benefit from careful editing. Many figures (particularly overlay plots) are qualitative and difficult to interpret quantitatively. The use of a Google Docs link for data sharing is not appropriate for long-term reproducibility. Last by not the least, some references are repeated unnecessarily.

The idea of leveraging operative temperature instead of ambient temperature is conceptually interesting. The attempt to connect ecological data to engineering design is commendable. The clustering results visually suggest increased granularity compared to air temperature. However, these strengths are not sufficient to offset the methodological weaknesses. The manuscript requires substantial revision before it can be considered for publication. At minimum, the authors must provide robust statistical and clustering validation, address data quality and sampling limitations, include mechanistic or physics-based validation of morphological relevance, clarify and temper overstated conclusions Without these improvements, the work remains conceptually interesting but scientifically unconvincing.

Reviewer #5: In this study, the authors tried to update a thermally oriented bioinspiration search method by clustering species spatial group locations based on growing season operative temperature distributions. Results show that clustering based on ambient air temperature yielded two broad regional groups, and margin analysis revealed that cooler climates generally correlate with increased aspect ratios and significantly decreased edge radii. Below lists some suggestions.

1, 73 digital leaf specimens are investigated. It would be better to give a brief introduction of these specimens. How you chose these specimens.

2, how many specimens selected in different regions.

3, the authors mentioned the thermal design, however, the results only show the relation between temperature and characteristic radii. Besides temperature, the thermal design may also relate to thermal environment like the wind, rain etc.

4, the statistical method, like T-test, used in this study should be described in detail.

Reviewer #6: The reviewer’s expertise is primarily in heat transfer and thermal systems. Therefore, the evaluation focuses on the thermophysical formulation and interpretation of the operative temperature concept, as well as its relevance for thermal design applications. Biological and morphological aspects are assessed at a general level.

The manuscript proposes the use of operative temperature as an alternative metric to ambient air temperature for identifying thermally driven morphological adaptations in biological systems. The idea of incorporating radiative and convective effects into a single environmental descriptor is conceptually interesting. However, from a heat transfer perspective, the methodological formulation, physical interpretation, and engineering relevance of the results require significant clarification.

Following should be taken into consideration:

1. The manuscript introduces operative temperature as a more comprehensive metric, but its physical meaning and limitations are not clearly discussed. The assumption of a “massless surface” used for its calculation is particularly problematic and requires justification. It remains unclear how well this metric represents real leaf thermodynamics, especially under transient and heterogeneous environmental conditions.

2. The study relies entirely on calculated operative temperatures without validation. How sensitive the operative temperature is to input parameters such as wind speed, radiation intensity, or surface properties?

Without such validation, it is difficult to assess whether the observed differences between clusters are physically meaningful.

3. The main argument is that operative temperature provides "greater granularity". However, increased clustering resolution alone does not demonstrate improved physical relevance. The manuscript does not convincingly show that operative temperature leads to better or more physically grounded interpretation of heat transfer phenomena.

4. The discussion of results remains largely qualitative and does not explicitly link observed trends to heat transfer mechanisms. While references are made to convection and diffusion processes, no quantitative analysis is provided.

From a thermal engineering perspective, the study does not establish how the identified differences influence heat transfer performance.

5. The observed variations in edge radius and aspect ratio are relatively small. The manuscript does not provide a clear analysis of whether these differences are significant from a heat transfer standpoint. Specifically, it would strengthen the paper considerably to address whether the observed differences in edge radius, e.g. in the order of 10–15% between groups, correspond to any meaningful change in convective heat transfer coefficient or Nusselt number. Without such an assessment, the connection between the biological findings and thermal engineering applications remains speculative.

**********

what does this mean?). If published, this will include your full peer review and any attached files.

If you choose “no”, your identity will remain anonymous but your review may still be made public.

**Do you want your identity to be public for this peer review?**  For information about this choice, including consent withdrawal, please see our Privacy Policy

Reviewer #1: No

Reviewer #2: No

Reviewer #3: No

Reviewer #4: **Yes:** Dr. Arjun Dey

Reviewer #5: No

Reviewer #6: No

**********

[NOTE: If reviewer comments were submitted as an attachment file, they will be attached to this email and accessible via the submission site. Please log into your account, locate the manuscript record, and check for the action link "View Attachments". If this link does not appear, there are no attachment files.]

To ensure your figures meet our technical requirements, please review our figure guidelines: https://journals.plos.org/plosone/s/figures

You may also use PLOS’s free figure tool, NAAS, to help you prepare publication quality figures: https://journals.plos.org/plosone/s/figures#loc-tools-for-figure-preparation.

NAAS will assess whether your figures meet our technical requirements by comparing each figure against our figure specifications.

# Author comment: Author response to Decision Letter 1

**DOI:** 10.1371/journal.pone.0358463.r002

29 Jun 2026

Subject: Response to Reviewers – [PONE-D-25-37512]

Date: June 18th, 2026

Dear Shailender and Reviewers,

Thank you for the in-depth and thorough review of our manuscript Beyond Air Temperature: An examination of leveraging operative temperature space for the discovery of bioinspired thermal design features. We have carefully reviewed the comments provided and have worked to address the points raised within the paper. Their insights have been invaluable in reassessing the methods of the study and have providing meaningful feedback that has strengthening the research presented.

In response to the revision decision, we have reworked the study to address previous shortcomings by incorporating improved statistical rigor and expanding the number of specimens incorporated in this work. A significant revision was the calculation of the edge radii parameter to focus on radii with respect to edge deviation rather than the leaf width. This pivot better reflects currently fielded edge structures shown in the newly incorporated Figure 4 and as cited in reference (40). These ‘undulations’ are largely used to propagate turbulent structures near the edges of surfaces as illustrated in (40) and additional references (41)(42)(43). This pivot better reflects the incorporation of this feature in enhancing edge transport in cooling designs.

Another consideration brought up by the reviewers is the applicability of the operative temperature as the output of a modeled leaf rather than a derived climate parameter. The operative temperature parameter developed is intended to serve as an abiotic factor to differentiate thermal environments without treating heat related forcings as completely independent drivers. It is intended to drive investigation along thermal clines rather than explicitly model physiological outcomes. A new subsection, Limits of the parameter, has been incorporated in the Discussion section to explicitly discuss the factor and its intended use for a study framework.

Additionally, an extra version of the manuscript has been uploaded this time to address the readability issues multiple reviewers cited by figure placement at the end of the document. This is intended to be the ‘as desired’ placement and size of figures within the text.

We have drafted point-by-point responses to the reviewers’ detailed remarks below. Thank you for consideration and feedback on our submission.

Sincerely,

Thomas Slavens

Corresponding Author

University of Oklahoma, College of Engineering

Email: tslavens@ou.edu

REVIEW COMMENTS AND RESPONSES– [PONE-D-25-37512]

Reviewer 1: Integrate Broader Literature Comparisons "The manuscript references key studies on leaf thermoregulation, but a more comprehensive comparison with similar herbarium-based analyses (e.g., those using digitized collections for trait evolution) would strengthen the novelty claim."

Author Response: A new section in the document was introduced covering the use of digital herbarium for this study. A number of references were introduced that used this relatively new digital resource to study species ranges and phenology.

Methods: “Study specimens from digital herbarium”

Reviewer 1: Refine the Statistical Analysis Approach "The current statistical analysis relies on independent Student’s T-tests to compare morphological parameters between multiple groups (e.g., comparing groups Ao, Bo, Co, and Do). Using multiple T-tests increases the risk of Type I errors (false positives). It is highly recommended to use an Analysis of Variance (ANOVA) followed by an appropriate post-hoc test (such as Tukey's HSD) for multiple comparisons. Additionally, please state whether the data met the assumptions of normality and homogeneity of variances (e.g., via Shapiro-Wilk and Levene's tests); if not, a non-parametric alternative like the Kruskal-Wallis test should be utilized given the varying and sometimes small sample sizes (e.g., n=9 for group Co)."

Author Response: The statistical assessments for the operative temperature groups have been refined and leverage an appropriate ANOVA and post hoc test to determine significant group differences.

Results: “Results of margin assessments on segregated leaf populations.

Reviewer 1: Enhance Figure Clarity and Accessibility "Figures such as 7 and 8 effectively illustrate trends in morphological parameters, but they could be improved for better readability. Consider adding color-coding or annotations to clearly distinguish between air temperature and operative temperature groupings (e.g., using distinct symbols or legends)."

Author Response: Figures have been revamped with higher resolution images along with tables with descriptive statistics of edge radii between groups. Further documentation of graphical information for leaf width and aspect ratio in tabular form to be included in supplemental data set.

Results: “Results of margin assessments on segregated leaf populations.

Reviewer 1: Enhance the Presentation of Statistical Results "While Figures 7 and 8 provide a good visual representation of the morphological trends, relying solely on bar charts with error bars makes it difficult for readers to extract precise values. Please consider adding a summary table that explicitly lists the sample size (n), mean, standard deviation, and precise p-values for aspect ratio, leaf width, and non-dimensionalized edge radii across all temperature groupings (both air and operative). This will greatly improve the clarity and rigor of the data presentation."

Author Response: Descriptive statistics and normality test results for edge radii have been included in Tables 1 & 2. Between group comparison statics information is included in updates within the results section.

Results: “Results of margin assessments on segregated leaf populations.” See updated Figures 11 & 12 and Tables 1 & 2.

Reviewer 2: The authors should make the connection between leaf-edge morphology and heat-transfer problems more explicit. At present, this connection is understandable, but it is not developed strongly enough. A short explanation is needed to show why the edge of a leaf is important from the viewpoint of boundary-layer behavior, local diffusion, and convective exchange. This would make the transition from botanical observation to bioinspired thermal design more convincing.

Author Response: Additional information on how the edge radii is related to prior industry work and studies have been added along with an additional figure highlighting the transition in edge serration with a change in radii parameter. Further, adjustment to the non-dimensionalization to be based on the average edge deviation, rather than leaf width, better associates this parameter with edge effect development. Further information was added that highlights studies on the aerodynamic stability and heat transfer impacts leading edge undulations provide (similar in form to the edge margin nodules on the leaf). While these do not directly tie in the radii to heat transfer rates, these do indicate that they have an impact to boundary layer dynamics on the adjacent surfaces.

Results: “Results of margin assessments on segregated leaf populations.” See updated Figures 11 & 12 and Tables 1 & 2.

Reviewer 2: The paper would benefit from a clearer separation of two different results. One result is methodological: operative temperature provides a more detailed and physically meaningful classification of the species range. Another result is morphological: the identified groups show differences in leaf-margin parameters, especially in the characteristic edge radius. These two results are connected, but they should be presented as two consecutive levels of evidence. This would make the logic of the paper clearer.

Author Response: Additional verbiage within the introduction section has been added to better parse the two results the study directly developed.

Methods: “Leaf margin extraction and analysis”

Reviewer 2: The selected DBSCAN search radius of 2.5 °C needs additional explanation. From a heat-transfer point of view, this value is not a direct physical temperature difference between two locations. The clustering is performed in a multidimensional temperature space that includes monthly maximum, minimum, and average temperatures. Therefore, the meaning of a 2.5 °C distance in this space is not self-evident. The authors should explain how this value was selected, whether the temperature variables were normalized or scaled before clustering, and whether the obtained groups remain stable if this parameter is moderately changed. Even a short sensitivity check would make the clustering results more reliable.

Author Response: The 2.5°C parameter was developed through a sensitivity assessment of both air and operative temperature spaces on the raw values from species basal locations. Results section has been updated to highlight this sensitivity study and identify how the 2.5°C parameter was established.

Introduction, last paragraph.

Reviewer 2: The limited number of leaf specimens should be discussed more clearly. This limitation is acceptable for a study that tests an idea, but it should be treated explicitly. Some operative-temperature groups contain relatively few specimens, and the material comes from herbarium images collected in different years and probably under different local growth conditions. The authors should comment on how specimen source, collection year, leaf selection, possible damage, leaf age, and local microenvironment may affect the interpretation of the statistical results.

Author Response: To help strengthen results, further specimens were included in the study where possible. Sans grouping Co, most groups have >20 specimens with their respective clusters. Co is limited by availability in digital herbarium databases and as such may present an incomplete view of species behavior in this location.

Results: “Results of cluster-based analysis on leaf specimen segregation”

Reviewer 2: The manuscript does not yet demonstrate a ready engineering solution. It identifies a candidate biological morphology that may be useful for further thermal or diffusive design studies. I do not consider this a serious weakness, because the paper is mainly methodological and exploratory. However, this level of evidence should be formulated clearly, so that the engineering meaning of the results can be stated carefully.

Author Response: The intent of the method is to allow engineers that are not explicitly engaged in biological or ecological studies an improved abiotic parameter to gauge environmental heat load a species may encounter. Once species are found to inhabit distinctly loaded locations, the intent would be to leverage taxa comparisons between regions to identify physiological or morphological adaptions of interest to model and evaluate further. A pre-exploratory notional configuration of interest based on this leaf edge undulations is cited in (40) and shown in new Figure 4.

Methods: “Leaf edge margin extraction and analysis”

Reviewer 3: First of all, this paper is a little bit confusing, since the authors do not clearly explain how the operative temperature is calculated.

Author Response: Details of the operative temperature calculations are captured in the authors prior work cited in reference (5).

Reviewer 3: Moreover, in the view of heat transfer, there should be some kind of assumptions when calculating such operative temperature. How could those assumptions affect the clustering analysis results?

Author Response: A major assumption of the operative temperature calculation is the magnitude of the surface width for convective heat transfer calculations. New Figure 1 shows a typical diurnal calculation of operative temperature for both a 3 cm and 5 cm surface. This adjustment shows an approximately 3°C increase in peak operative temperature. The 3cm characteristic length was ultimately chosen due to (1) better approximation of transport mechanisms along leaf edges and (2) to ensure that the flow modeled would be within the laminar Reynolds number of the calculation under large swings in ambient wind speeds.

Methods: “Species subgroup development based on temperature space”

Reviewer 3: Lastly, the current version is more like a botanical paper in my view, and how would these finds by operative temperature be utilized for bioinspired thermal design ? Please give a practical example at least.

Author Response: The intent of this work is to help bridge gaps between biological disciplines and engineering design. Much of the focus of the study is to provide a framework to allow a thermal designer to investigate where a species of interest may be identified by providing a singular parameter that encompasses more of the heat loadings a species may encounter. In developing this framework, the bulk of the research has been centered on developing and interrogating the operative temperature parameter in providing a context from which further morphological studies may be launched. A pre-exploratory notional configuration of interest based on this leaf edge undulations is cited in (40) and shown in new Figure 4.

Methods: “Leaf edge margin extraction and analysis”

Reviewer 4: The manuscript repeatedly suggests that operative temperature enables identification of thermally relevant morphological features for engineering design. However, the study demonstrates only weak correlations between cluster assignments and simple geometric descriptors. There is no demonstration that the identified features (e.g., reduced edge radii) meaningfully affect heat transfer performance. The conclusion that observed trends relate to early-season thermal adaptation rather than peak heat load is speculative. In its current form, the manuscript does not establish a causal or mechanistic link between operative temperature and functional morphology.

Author Response: The intent of the study is to demonstrate the operative temperature parameter as a metric of merit in helping to frame the aggressiveness of heat load a taxa may encounter in the field. The use of the margin assessment of a distributed species is intended to gauge the effectiveness of this parameter in comparison to using air temperature alone. Additional explanation of prior studies and industrial development of convective apparatus leveraging leading edge radii augmentation has been provided included in the methods section.

With the updated analysis and results, the authors have adjusted the observations and conclusions to support further studies down cold-weather dynamics of impacts to leaf margin serration as supported by Figures 9, 14, and 15. From Figure 9, the scatter plot illustrates Maximum To overlap between groups Bo and Ao, and between groups Bo and Co. In Figures 14 and 15, it is observed that differences in temperatures between the clusters is only observed in early months between Bo and Ao. These results would not support that the trend of reducing margin edge radii would be strongly driven by mid-season peak temperatures.

Discussion: “Margin assessments of P. heterophylla samples”

Conclusions.

Reviewer 4: The analysis is based on 73 manually selected leaves, drawn from heterogeneous herbarium sources spanning long time periods and unknown environmental conditions. The dataset is small relative to the variability inherent in biological morphology. No control exists for confounding variables (age, hydration state, light exposure, damage). The authors explicitly acknowledge these inconsistencies but proceed without addressing their impact quantitatively. This raises serious concerns that the reported trends may reflect sampling artifacts rather than biological signal.

Author Response: Additional specimens (103) and updated descriptions of the controls of the specimens to account for leaf maturity and collection are provided in a new methods section specifically on the utilization of herbarium species for this study.

Methods: “Study specimens from digital herbarium”

Reviewer 4: Leaf outlines are manually digitized and resampled however; no validation of repeatability or inter-operator consistency is provided. The selection of “clean” leaves introduces subjective bias. The downstream metric (edge radius) is highly sensitive to boundary definition. Without uncertainty

Attachment Submitted filename: PLOSOne_Slavens_BeyondAmbientAirTemp_ResponseDocument.docx

# Peer review: Decision Letter 1

**DOI:** 10.1371/journal.pone.0358463.r003

**Authors:** Mohammad Zuber

23 Jul 2026

Dear Dr. Slavens,

==============================

==============================

Please submit your revised manuscript by Sep 06 2026 11:59PM. If you will need more time than this to complete your revisions, please reply to this message or contact the journal office at plosone@plos.org. When you're ready to submit your revision, log on to https://www.editorialmanager.com/pone/ and select the 'Submissions Needing Revision' folder to locate your manuscript file.

- A letter that responds to each point raised by the academic editor and reviewer(s). You should upload this letter as a separate file labeled 'Response to Reviewers'.
- A marked-up copy of your manuscript that highlights changes made to the original version. You should upload this as a separate file labeled 'Revised Manuscript with Track Changes'.
- An unmarked version of your revised paper without tracked changes. You should upload this as a separate file labeled 'Manuscript'.

If you would like to make changes to your financial disclosure, please include your updated statement in your cover letter. Guidelines for resubmitting your figure files are available below the reviewer comments at the end of this letter.

If applicable, we recommend that you deposit your laboratory protocols in protocols.io to enhance the reproducibility of your results. Protocols.io assigns your protocol its own identifier (DOI) so that it can be cited independently in the future. For instructions see: https://journals.plos.org/plosone/s/submission-guidelines#loc-laboratory-protocols. Additionally, PLOS ONE offers an option for publishing peer-reviewed Lab Protocol articles, which describe protocols hosted on protocols.io. Read more information on sharing protocols at https://plos.org/protocols?utm_medium=editorial-email&utm_source=authorletters&utm_campaign=protocols.

As the corresponding author, your ORCID iD is verified in the submission system and will appear in the published article. PLOS supports the use of ORCID, and we encourage all coauthors to register for an ORCID iD and use it as well. Please encourage your coauthors to verify their ORCID iD within the submission system before final acceptance, as unverified ORCID iDs will not appear in the published article. *Only*  the individual author can complete the verification step; PLOS staff *cannot*  verify ORCID iDs on behalf of authors.

We look forward to receiving your revised manuscript.

Kind regards,

Mohammad Zuber

Academic Editor

PLOS One

Journal Requirements:

If the reviewer comments include a recommendation to cite specific previously published works, please review and evaluate these publications to determine whether they are relevant and should be cited. There is no requirement to cite these works unless the editor has indicated otherwise.

[Note: HTML markup is below. Please do not edit.]

Reviewers' comments:

Reviewer's Responses to Questions

**Comments to the Author**

Reviewer #1: All comments have been addressed

Reviewer #2: (No Response)

Reviewer #5: All comments have been addressed

Reviewer #6: All comments have been addressed

**********

2. Is the manuscript technically sound, and do the data support the conclusions?

Reviewer #1: Yes

Reviewer #2: Partly

Reviewer #5: Yes

Reviewer #6: Yes

**********

3. Has the statistical analysis been performed appropriately and rigorously?

Reviewer #1: Yes

Reviewer #2: No

Reviewer #5: Yes

Reviewer #6: Yes

**********

4. Have the authors made all data underlying the findings in their manuscript fully available?

The PLOS Data policy

Reviewer #1: Yes

Reviewer #2: Yes

Reviewer #5: Yes

Reviewer #6: Yes

**********

5. Is the manuscript presented in an intelligible fashion and written in standard English?

Reviewer #1: Yes

Reviewer #2: No

Reviewer #5: Yes

Reviewer #6: Yes

**********

Reviewer #1: Small advice to author: The authors did not follow your statistical recommendation perfectly for the two-group comparison (Aa vs Ba). They retained an independent t-test there, but statistically that is completely acceptable because only two groups are being compared. Your concern was primarily about performing many pairwise t-tests across four groups, and they corrected exactly that by switching to ANOVA with Tukey HSD for the operative-temperature analysis. So I would not press this point further.

The authors have responded satisfactorily to my comments. The manuscript has been substantially improved, particularly with respect to the statistical analysis, inclusion of descriptive statistics, expansion of the literature review, and improved figure presentation. I appreciate the authors' efforts in revising the manuscript and have no further major concerns. I RECOMMEND ACCEPTANCE.

Reviewer #2: The revised manuscript is substantially better, and the authors clearly did serious work. The paper is now much easier to follow and, in my opinion, it is no longer between major revision and rejection. It is rather between a strong revision and possible acceptance.

Several improvements should be recognized.

+ The connection between leaf-edge morphology and heat-transfer problems is now much clearer. The added discussion of boundary-layer behavior, vortical structures, flow stability, and engineered edge undulations makes the bioinspired interpretation more convincing. The new schematic is also useful. The paper still does not give a ready engineering solution, but now it gives a reasonable candidate for future CFD and experimental work, which is sufficient for an exploratory study.

+ The manuscript also separates better the two main results: environmental clustering and morphological comparison. This was not fully clear before, but now the logic is much more readable.

+ The herbarium part is improved considerably. The number of specimens increased from 73 to 103, about 41%. The authors now describe the selection criteria: more than 90% preserved leaf margin, leaf width of 6-16 cm, broad geographic coverage, and collection across the growing season. The new discussion of herbarium biases is also useful and honest.

+ The statistical approach is improved in principle. ANOVA and post-hoc comparisons are more appropriate than multiple independent t-tests. The added sample sizes, descriptive statistics, and normality tests are welcome.

+The operative-temperature method is also described more clearly. The manuscript now explains that clustering uses maximum, minimum, and average temperatures for 8 months, from March to October, therefore 24 dimensions in total. The comparison of 3 cm and 5 cm characteristic lengths and the approximately 3°C difference in peak operative temperature are useful additions. The comparison with a low-mass physical model is also a good step.

+ The new limitations section is important. It now becomes clear that operative temperature is an environmental screening parameter, not the actual physiological temperature of a leaf. The authors also correctly mention that transpiration is not included.

Finally, placing figures near the relevant text makes the manuscript much more comfortable to read. This sounds like a small thing, but here the figures are half of the argument, so it is not a small thing.

However, three scientific issues still need attention.

1. Robustness of the DBSCAN result

This is the main remaining problem.

The clustering is performed in a 24-dimensional space using raw, unscaled temperature values. Operative temperature has a wider numerical range than 2 m air temperature, but the same DBSCAN radius, ε = 2.5°C, is used for both spaces.

The authors obtain 2 clusters in air-temperature space and 4 clusters in operative-temperature space. However, a more dispersed point cloud can naturally produce more clusters with the same fixed ε. DBSCAN, unfortunately, does not know that one space is more physically meaningful than another.

Therefore, the authors should show that the result is not mainly a scaling effect. At minimum, they should:

• repeat clustering after standardization of the 24 variables, or quantitatively justify why the same absolute ε is appropriate;

• report the number and percentage of noise points;

• show stability under moderate changes around ε = 2.5°C;

• clarify whether minPts = 30 was fixed and whether a modest change of this value affects the result.

The model sensitivity also deserves a short comment. Changing the characteristic length from 3 cm to 5 cm changes peak operative temperature by about 3°C, which is already larger than ε = 2.5°C. It is not necessary to repeat the whole study, but the possible effect on cluster boundaries should be discussed.

Without this check, the central 2-versus-4 cluster result remains not fully safe.

2. Accounting for specimens and checking statistics

The manuscript states that 103 specimens were analyzed.

For the air-temperature groups, the reported sample sizes are 67 + 25 = 92. Thus, 11 specimens are absent from this analysis.

For the operative-temperature groups, the sample sizes are 24 + 24 + 11 + 25 = 84. Thus, 19 specimens are absent.

Probably these specimens belong to DBSCAN noise, but the leaves should not disappear silently. The manuscript should state clearly:

• how many specimens were assigned to every cluster;

• how many were excluded as noise;

• whether there were other exclusions;

• whether the air-temperature and operative-temperature analyses use different subsets.

Because the two analyses use 92 and 84 specimens, respectively, part of their difference may come from sample composition. A comparison using the common subset assigned in both analyses would be useful.

The statistical tables also need careful checking.

The aspect-ratio means for the operative-temperature groups are reported as 7.21, 6.21, 6.47, and 4.95. These values appear to duplicate the edge-radius values and are inconsistent with the figure, where aspect ratio is around 0.8.

The manuscript also says that all groups satisfied normality, but at least two Anderson-Darling results have p = 0.04. With alpha = 0.05, this is not normality confirmation.

The authors should verify all sample sizes, means, standard deviations, test statistics, degrees of freedom, and p-values. Exact p-values, effect sizes, and confidence intervals for the main comparisons would be preferable.

3. The conclusions are still a little more enthusiastic than the data

The authors are perhaps a little too enthusiastic here. The data show an interesting association, but they do not yet demonstrate the proposed mechanism. It also shows that operative-temperature space can produce 4 environmental groups instead of 2 under the selected DBSCAN settings.

It does not directly prove that:

• operative temperature caused the morphology;

• the morphology is specifically a heat-transfer adaptation;

• cool-season conditions are the causal driver;

• summer heat load is not important;

• the measured radius produces an engineering heat-transfer benefit.

The strongest morphological difference is mainly between the most separated groups, Ao and Do. The intermediate groups Bo and Co are not consistently distinguished by the main edge-radius parameter. In addition, the air-temperature grouping already gives a clear morphological difference.

Therefore, the manuscript demonstrates greater environmental granularity more convincingly than greater morphological discrimination.

Terms such as “heat-transfer-based morphological adaptations” should be replaced by more careful wording, for example “candidate morphological features associated with different regional thermal environments.” The cool-season interpretation should be presented as a hypothesis consistent with the results, not as an established mechanism.

Technical preparation

Before resubmission, the authors should update all fields and cross-references in the Word document and then inspect both the Word and PDF versions.

At present, some figures have duplicated references, one valid and one inherited from the previous version. After field updating, these produce messages such as:

Error: Reference source not found.

All obsolete fields, invalid figure references, duplicated cross-references, and old manuscript identifiers should be removed or corrected.

Word fields should not be allowed to make independent scientific decisions in the final PDF.

Overall, I recommend Major Revision. This is not the same situation as in the first round. The manuscript is now potentially publishable, and many previous concerns were addressed well. However, the clustering robustness, accounting of all 103 specimens, statistical consistency, and strength of conclusions should be corrected before acceptance.

-----------------

Remarks below are not mandatory, just for further directions

Optional suggestion concerning the leaf-margin parameter. The authors may consider adding one more simple dimensionless descriptor of leaf-margin complexity.

If only one parameter should be selected, I would probably use the normalized excess edge length:

E = (Praw - Psmooth) / Psmooth

where Praw is the length of the actual leaf margin and Psmooth is the length of the corresponding smoothed margin. This parameter is easy to interpret: E = 0 represents a perfectly smooth edge, while E = 0.10 means that serration adds 10% to the edge length. It is dimensionless, largely independent of overall leaf size, and can be calculated directly from the raw and NURBS-smoothed contours already available in the present analysis. It may therefore provide a useful and rather transparent check of the proposed characteristic-radius parameter.

However, from the heat-transfer viewpoint, one parameter may be slightly optimistic. Two margins can have the same excess length but very different structures: one may contain many small teeth, while another contains only several large undulations. These geometries are unlikely to interact with the boundary layer in the same manner. If the authors wish to connect the morphology more directly with the physical mechanism proposed in the paper, a compact set of dimensionless quantities could be more informative:

A = RMS (root mean square) edge deviation / leaf width

L = characteristic tooth spacing / leaf width

S = RMS edge deviation / characteristic tooth spacing

Here, A represents the relative amplitude of the edge undulations, L represents their relative wavelength or spacing, and S represents their characteristic steepness. The last parameter may be particularly relevant to local flow separation and vortex generation.

I do not suggest that the authors replace or extend the present analysis as part of the current revision. These descriptors may rather be useful as a direction for future work, particularly if the authors continue toward a more direct comparison between leaf-edge geometry and boundary-layer or heat-transfer behavior. The normalized excess edge length could provide a simple general measure of margin complexity, while relative amplitude, spacing, and steepness may help to distinguish geometries that have similar overall serration but potentially different flow effects.

Reviewer #5: (No Response)

Reviewer #6: All questions and doubts have been explained and addressed. No furter action is required. The paper is now ready to be accepted

**********

what does this mean?). If published, this will include your full peer review and any attached files.

If you choose “no”, your identity will remain anonymous but your review may still be made public.

**Do you want your identity to be public for this peer review?**  For information about this choice, including consent withdrawal, please see our Privacy Policy

Reviewer #1: No

Reviewer #2: No

Reviewer #5: No

Reviewer #6: No

**********

[NOTE: If reviewer comments were submitted as an attachment file, they will be attached to this email and accessible via the submission site. Please log into your account, locate the manuscript record, and check for the action link "View Attachments". If this link does not appear, there are no attachment files.]

To ensure your figures meet our technical requirements, please review our figure guidelines: https://journals.plos.org/plosone/s/figures

You may also use PLOS’s free figure tool, NAAS, to help you prepare publication quality figures: https://journals.plos.org/plosone/s/figures#loc-tools-for-figure-preparation.

NAAS will assess whether your figures meet our technical requirements by comparing each figure against our figure specifications.

# Author comment: Author response to Decision Letter 2

**DOI:** 10.1371/journal.pone.0358463.r004

24 Aug 2026

--------------------------------------------------

Reviewer 1: Small advice to author: The authors did not follow your statistical recommendation perfectly for the two-group comparison (Aa vs Ba). They retained an independent t-test there, but statistically that is completely acceptable because only two groups are being compared. Your concern was primarily about performing many pairwise t-tests across four groups, and they corrected exactly that by switching to ANOVA with Tukey HSD for the operative-temperature analysis. So I would not press this point further.

The authors have responded satisfactorily to my comments. The manuscript has been substantially improved, particularly with respect to the statistical analysis, inclusion of descriptive statistics, expansion of the literature review, and improved figure presentation. I appreciate the authors' efforts in revising the manuscript and have no further major concerns. I RECOMMEND ACCEPTANCE.

Response: Thank you for the time to review our paper and the feedback.

--------------------------------------------------

Reviewer 2: Robustness of the DBSCAN result

This is the main remaining problem.

The clustering is performed in a 24-dimensional space using raw, unscaled temperature values. Operative temperature has a wider numerical range than 2 m air temperature, but the same DBSCAN radius, ε = 2.5°C, is used for both spaces.

The authors obtain 2 clusters in air-temperature space and 4 clusters in operative-temperature space. However, a more dispersed point cloud can naturally produce more clusters with the same fixed ε. DBSCAN, unfortunately, does not know that one space is more physically meaningful than another.

Therefore, the authors should show that the result is not mainly a scaling effect. At minimum, they should:

• repeat clustering after standardization of the 24 variables, or quantitatively justify why the same absolute ε is appropriate;

• report the number and percentage of noise points;

• show stability under moderate changes around ε = 2.5°C;

• clarify whether minPts = 30 was fixed and whether a modest change of this value affects the result.

The model sensitivity also deserves a short comment. Changing the characteristic length from 3 cm to 5 cm changes peak operative temperature by about 3°C, which is already larger than ε = 2.5°C. It is not necessary to repeat the whole study, but the possible effect on cluster boundaries should be discussed.

Without this check, the central 2-versus-4 cluster result remains not fully safe. The authors thank Reviewer 2 for the further insight in utilization of the DBSCAN method in identifying clusters in temperature spaces.

Response: In reflection of utilization of the DBSCAN method, the authors have pivoted to adjust scan radii to be individually tailored to each temperature dimensional space rather than a single target value based. As pointed out by the reviewer, the DBSCAN is sensitive to the scale of the space it is executed within. Since this study is focused on the ability of the inclusion of a heat flux balance to broaden the temperature space of a species to identify inter-species variations of interest, it did make more sense to alter the DBSCAN search radius to each temperature space rather than non-dimensionalize spaces to parity. The new search radii were based on 90% capture of the species points and supported by the factors position in a k-distance graph. Sensitivity to neighbor count is also provided with the selected neighbor count being chosen as 35 for both temperature spaces. Additionally, a sensitivity to the chosen ε values were conducted for both air and operative temperature spaces showing a moderate robustness of ~0.1°C for air and 0.15°C for operative space.

A final paragraph was added to address the magnitude impacts of changes in leaf width assumptions relative to shifts in temperature space. As observed in Fig. 1, increase in lc pushes the maximum temperature calculated due to the increase in received solar radiation to the surface and the reduced convective transfer over a larger flat plate. This shift in peak could ultimately provide larger differences across population areas increasing group resolution from the cluster analysis. A reduction in lc would work opposite to this keeping partitions closer to as observed in air temperature space. A further study of a species with relatively thin leaves (willow or pine) and wide leaves (maple) would be of interest to provide insight into this relationship. Figure 5

Supplement 3, Figures 1, 2, 3, and 4

Discussion section, “Limitations of the parameter”, last paragraph

--------------------------------------------------

Reviewer 2: Accounting for specimens and checking statistics

The manuscript states that 103 specimens were analyzed.

For the air-temperature groups, the reported sample sizes are 67 + 25 = 92. Thus, 11 specimens are absent from this analysis.

For the operative-temperature groups, the sample sizes are 24 + 24 + 11 + 25 = 84. Thus, 19 specimens are absent.

Probably these specimens belong to DBSCAN noise, but the leaves should not disappear silently. The manuscript should state clearly:

• how many specimens were assigned to every cluster;

• how many were excluded as noise;

• whether there were other exclusions;

• whether the air-temperature and operative-temperature analyses use different subsets.

Because the two analyses use 92 and 84 specimens, respectively, part of their difference may come from sample composition. A comparison using the common subset assigned in both analyses would be useful.

The statistical tables also need careful checking.

The aspect-ratio means for the operative-temperature groups are reported as 7.21, 6.21, 6.47, and 4.95. These values appear to duplicate the edge-radius values and are inconsistent with the figure, where aspect ratio is around 0.8.

The manuscript also says that all groups satisfied normality, but at least two Anderson-Darling results have p = 0.04. With alpha = 0.05, this is not normality confirmation.

The authors should verify all sample sizes, means, standard deviations, test statistics, degrees of freedom, and p-values. Exact p-values, effect sizes, and confidence intervals for the main comparisons would be preferable. The full 103 represented the total number of digitized specimens. Clarity has been added to the text to show how this population was reduced first by leaf width standards to ensure mature and representative leaves. Subsequent reductions were then made based on the occurrence of the specimen within an identified temperature-driven spatial cluster region. This evolution is highlighted in new Figure 12 and addressed in the results and discussion sections.

Response: A secondary assessment was performed to address inconsistencies of results of the Na = 98 of the air temperature groupings versus the No = 86 of the operative temperature groupings. This study repeated the assessment of the air temperature group, but excluded those specimens that were not used in the operative temperature assessment. Results are included in supplement 3 and are consistent with the full Na = 98 analysis.

Tables have been updated to represent current population assessments and checked for consistency with figures. With the exception of air groupings leaf width, all Anderson-Darling groups are >= 0.5. For the exception (air groupings leaf width comparison), a non-parametric test is used to compare these groups (Wilcoxon rank sum test). Figure 12, Supplementary Figure 5 and supplementary Table 1.

--------------------------------------------------

Reviewer 2: The conclusions are still a little more enthusiastic than the data

The authors are perhaps a little too enthusiastic here. The data show an interesting association, but they do not yet demonstrate the proposed mechanism. It also shows that operative-temperature space can produce 4 environmental groups instead of 2 under the selected DBSCAN settings.

It does not directly prove that:

• operative temperature caused the morphology;

• the morphology is specifically a heat-transfer adaptation;

• cool-season conditions are the causal driver;

• summer heat load is not important;

• the measured radius produces an engineering heat-transfer benefit.

The strongest morphological difference is mainly between the most separated groups, Ao and Do. The intermediate groups Bo and Co are not consistently distinguished by the main edge-radius parameter. In addition, the air-temperature grouping already gives a clear morphological difference.

Therefore, the manuscript demonstrates greater environmental granularity more convincingly than greater morphological discrimination.

Terms such as “heat-transfer-based morphological adaptations” should be replaced by more careful wording, for example “candidate morphological features associated with different regional thermal environments.” The cool-season interpretation should be presented as a hypothesis consistent with the results, not as an established mechanism.

Response: Conclusions, both within the abstract and the conclusion section have been couched to the explicit results. The conclusions are focused on the intent of the study to demonstrate the effectivity of population segregation in operative temperature space provides a higher descriptive framework than use of air temperature alone.

Further work, as discussed in the “Limitations of the Parameter” section, will be necessary in the future to refine the parameter use and correlate to other studies to help support thermal load driven claims of morphological adaptions. Revised Abstract and Conclusion sections.

Edit to the Discussion section “Margin assessments of P. heterophylla samples”, paragraph 4.

“Limitations of the Parameter”, paragraph 1.

--------------------------------------------------

Reviewer 2: Technical preparation

Before resubmission, the authors should update all fields and cross-references in the Word document and then inspect both the Word and PDF versions.

At present, some figures have duplicated references, one valid and one inherited from the previous version. After field updating, these produce messages such as:

Error: Reference source not found.

All obsolete fields, invalid figure references, duplicated cross-references, and old manuscript identifiers should be removed or corrected.

Word fields should not be allowed to make independent scientific decisions in the final PDF.

Response: Fields have been updated for final version; tracked changes version should be only for author review and the final clean version is also provided to ensure intended status of the revision is documented. See “PLOSOne_BeyondAmbientAirTemp_Revision-2-Manuscript_w-Figures.docx”

--------------------------------------------------

Reviewer 6: All questions and doubts have been explained and addressed. No further action is required. The paper is now ready to be accepted. Thank you for the time to review our paper and the feedback.

Attachment Submitted filename: PLOSOne_BeyondAmbientAirTemp_Revision2_ResponseDocument.docx

# Editor report: Decision Letter 2

**DOI:** 10.1371/journal.pone.0358463.r005

**Authors:** Mohammad Zuber

1 Sep 2026

Beyond air temperature: An examination of leveraging operative temperature space for the discovery of bioinspired thermal design features

PONE-D-26-12989R2

Dear Dr. Slavens,

We’re pleased to inform you that your manuscript has been judged scientifically suitable for publication and will be formally accepted for publication once it meets all outstanding technical requirements.

Within one week, you’ll receive an e-mail detailing the required amendments. When these have been addressed, you’ll receive a formal acceptance letter and your manuscript will be scheduled for publication.

An invoice will be generated when your article is formally accepted. Please note, if your institution has a publishing partnership with PLOS and your article meets the relevant criteria, all or part of your publication costs will be covered. Please make sure your user information is up-to-date by logging into Editorial Manager at Editorial Manager® and clicking the ‘Update My Information' link at the top of the page. For questions related to billing, please contact billing support.

If your institution or institutions have a press office, please notify them about your upcoming paper to help maximize its impact. If they’ll be preparing press materials, please inform our press team as soon as possible -- no later than 48 hours after receiving the formal acceptance. Your manuscript will remain under strict press embargo until 2 pm Eastern Time on the date of publication. For more information, please contact onepress@plos.org.

Kind regards,

Mohammad Zuber

Academic Editor

PLOS One

Additional Editor Comments (optional):

Reviewers' comments:

# Editor report: Acceptance letter

**DOI:** 10.1371/journal.pone.0358463.r006

**Authors:** Mohammad Zuber

PONE-D-26-12989R2

PLOS One

Dear Dr. Slavens,

I'm pleased to inform you that your manuscript has been deemed suitable for publication in PLOS One. Congratulations! Your manuscript is now being handed over to our production team.

At this stage, our production department will prepare your paper for publication. This includes ensuring the following:

* All references, tables, and figures are properly cited

* All relevant supporting information is included in the manuscript submission,

* There are no issues that prevent the paper from being properly typeset

You will receive further instructions from the production team, including instructions on how to review your proof when it is ready. Please keep in mind that we are working through a large volume of accepted articles, so please give us a few days to review your paper and let you know the next and final steps.

Lastly, if your institution or institutions have a press office, please let them know about your upcoming paper now to help maximize its impact. If they'll be preparing press materials, please inform our press team within the next 48 hours. Your manuscript will remain under strict press embargo until 2 pm Eastern Time on the date of publication. For more information, please contact onepress@plos.org.

You will receive an invoice from PLOS for your publication fee after your manuscript has reached the completed accept phase. If you receive an email requesting payment before acceptance or for any other service, this may be a phishing scheme. Learn how to identify phishing emails and protect your accounts at https://explore.plos.org/phishing.

If we can help with anything else, please email us at customercare@plos.org.

Thank you for submitting your work to PLOS One and supporting open access.

Kind regards,

PLOS One Editorial Office Staff

on behalf of

Dr. Mohammad Zuber

Academic Editor

PLOS One
